# Functional classification of protein structures by local structure matching in graph representation

**DOI:** 10.1002/pro.3416

**Published:** 2018-04-27

**Authors:** Caitlyn L. Mills, Rohan Garg, Joslynn S. Lee, Liang Tian, Alexandru Suciu, Gene D. Cooperman, Penny J. Beuning, Mary Jo Ondrechen

**Affiliations:** ^1^ Department of Chemistry and Chemical Biology Northeastern University Boston Massachusetts; ^2^ College of Computer and Information Science Northeastern University Boston Massachusetts; ^3^ Department of Mathematics Northeastern University Boston Massachusetts; ^4^ Present address: Joslynn S. Lee's current address: Howard Hughes Medical Institute, 4000 Jones Bridge Road Chevy Chase MD 20815‐6789

**Keywords:** protein function annotation, Graph Representation of Active Sites for Prediction of Function (GRASP‐Func), Structurally Aligned Local Sites of Activity (SALSA), Ribulose Phosphate Binding Barrel (RPBB) superfamily, 6‐Hairpin Glycosidase (6‐HG) superfamily, Concanavalin A‐like Lectins/Glucanase (CAL/G) superfamily

## Abstract

As a result of high‐throughput protein structure initiatives, over 14,400 protein structures have been solved by Structural Genomics (SG) centers and participating research groups. While the totality of SG data represents a tremendous contribution to genomics and structural biology, reliable functional information for these proteins is generally lacking. Better functional predictions for SG proteins will add substantial value to the structural information already obtained. Our method described herein, Graph Representation of Active Sites for Prediction of Function (GRASP‐Func), predicts quickly and accurately the biochemical function of proteins by representing residues at the predicted local active site as graphs rather than in Cartesian coordinates. We compare the GRASP‐Func method to our previously reported method, Structurally Aligned Local Sites of Activity (SALSA), using the Ribulose Phosphate Binding Barrel (RPBB), 6‐Hairpin Glycosidase (6‐HG), and Concanavalin A‐like Lectins/Glucanase (CAL/G) superfamilies as test cases. In each of the superfamilies, SALSA and the much faster method GRASP‐Func yield similar correct classification of previously characterized proteins, providing a validated benchmark for the new method. In addition, we analyzed SG proteins using our SALSA and GRASP‐Func methods to predict function. Forty‐one SG proteins in the RPBB superfamily, nine SG proteins in the 6‐HG superfamily, and one SG protein in the CAL/G superfamily were successfully classified into one of the functional families in their respective superfamily by both methods. This improved, faster, validated computational method can yield more reliable predictions of function that can be used for a wide variety of applications by the community.

**Abbreviations**
6‐HG: 6‐Hairpin Glycosidase
AGG: 1,4‐α‐l‐glucan glucohydrolase
ALF/ALG: 1,2‐α‐l‐fucosidase and α‐l‐galactosidase
ALR: α‐l‐rhamnosidase
ALY: lyases
AMAN: exo‐α‐1,6‐mannosidase
AMY: α‐amylase
CAL/G: Concanavalin A‐like Lectin/Glucanase
CBH: cellobiohydrolases
CDP: phosphorylase I
CELL: cellulases
EXC: endoglucanase/xylanase/chitosanase
ENDO: endoglucanases
GH16: GH family 16
GRASP‐Func: Graph Representation of Active Sites for Prediction of Function
HisA: phosphoribosylformimino‐5‐aminoimidazole carboxamide ribotide isomerase
HisF: imidazoleglycerolphosphate synthase
HPS: hexulose phosphate synthase
IGPS: indole‐3‐glycerol phosphate synthase
KGPDC: keto‐3‐gulonate‐phosphate decarboxylase
NAE: *N*‐acylglucosamine‐2‐epimerase
NGP: phosphorylase II
OMPDC: orotidine 5′‐monophosphate decarboxylase
PDB: Protein Data Bank
POOL: Partial Order Optimum Likelihood
PEP: peptidases
PRAI: phosphoribosyl anthranilate isomerase
RPBB: Ribulose Phosphate Binding Barrel
RPE: ribulose‐phosphate 3‐epimerase
SALSA: Structurally Aligned Local Sites of Activity
SG: Structural Genomics
TRE: trehalase
TrpA: tryptophan synthase
UGH: unsaturated glucuronyl hydrolase
URH: unsaturated rhamnogalacturonyl hydrolase
XYL: xylanases.

## Introduction

A wealth of new protein structures has been reported by structural genomics (SG) initiatives since 2000, but determination of the biochemical function of these structures has proved to be much more difficult than originally envisioned. Reliable methods for prediction of the function of proteins from their three‐dimensional (3D) structures constitute a critical current need; such capability will add tremendous value to SG data and advance significantly our understanding of protein function at the atomic level. While structural genomics holds tremendous promise for future applications of great benefit to society, a key step toward the realization of its (still largely untapped) full potential is the ability to determine the function of the thousands of protein structures for which the biochemical function is currently unknown or uncertain.

Current methods for assigning biochemical function are generally informatics based; sequence and structure comparisons are made between the query protein and other proteins in large databases, and functional assignments are transferred based on sequence or structure similarity with previously annotated proteins. Such methods have been described in recent reviews and compilations.^1^, ^2^, ^3^, ^4^, ^5^, ^6^, ^7^, ^8^, ^9^ Simple transfer of function based on global sequence or structure similarity can lead to misannotations.^10^, ^11^ Automated methods for functional annotation can cause misannotation errors to propagate through databases. Although important efforts are underway to assign correct functions to proteins,^12^ there are still thousands of protein structures without functional annotations and many more are misannotated.^13^

A local‐structure based function prediction method, Structurally Aligned Local Sites of Activity (SALSA), has been described recently.^4^, ^9^, ^14^, ^15^ SALSA establishes local spatial arrays of predicted functionally active residues for sets of proteins of known, experimentally determined biochemical function. A distinctive feature of the SALSA approach is that functionally active residues for each protein structure are predicted from computed chemical and electrostatic properties using Partial Order Optimum Likelihood (POOL),^16^, ^17^, ^18^ a machine learning method that predicts catalytically important residues using the structure of the query protein as the input. Predicted residues of common type in aligned spatial positions across a set of proteins of known, common function defines a Chemical Signature for that functional type. SALSA then matches the predicted functionally active residues for a protein of unknown function to the Chemical Signatures; a strong match of residue types in aligned spatial positions suggests that function may be transferred reliably.

In this work, a new approach to the local structure matching, Graph Representation of Active Sites for Prediction of Function (GRASP‐Func), is introduced; instead of using a Cartesian coordinate representation of the active site residues and relying on global multiple structure alignments as was done previously,^14^, ^15^, ^19^ the predicted sets of active residues are expressed in a topological graph representation. This enables much faster alignment and matching of the local active site structures. The Ribulose Phosphate Binding Barrel (RPBB), 6‐Hairpin Glycosidase (6‐HG), and Concanavalin A‐like Lectin/Glucanase (CAL/G) superfamilies are analyzed to illustrate application of the method and to make function predictions for some of the SG proteins predicted to be members of these superfamilies. Each superfamily was chosen for this study because it is medium‐sized with functional diversity and with generally good structural coverage and experimental functional characterization within each of the known functional families.

The RPBB superfamily (SCOP^20^ ID 51366) has a (β/α)‐barrel fold consisting of an eight‐stranded parallel β barrel surrounded by eight α helices.^21^ RPBB enzymes play essential roles in a variety of different metabolic pathways, including amino acid biosynthesis, pyrimidine biosynthesis, carbon fixation in plants, the nonoxidative phase of the pentose phosphate pathway (which generates ribose 5‐phosphate, a precursor for the biosynthesis of nucleotides), l‐ascorbate metabolism, and the ribulose‐monophosphate cycle. Some members of this superfamily also represent potential novel therapeutic targets for antibacterial or antifungal agents.^22^, ^23^, ^24^

The 6‐HG superfamily (SCOP ID 48208) contains all‐α structures sharing a common (α/α)_6_‐barrel fold. These enzymes share a similar catalytic mechanism, catalyzing the hydrolysis of glycosidic linkages in poly‐ or oligo‐saccharides. The CAL/G superfamily (SCOP ID 49899) contains all‐β proteins sharing a common antiparallel β‐strand sandwich core. These enzymes are involved in biosynthesis, cellular development, and localization, and other metabolic processes. Members of both the 6‐HG and CAL/G superfamilies have potential applications in biomass degradation and biofuel production. These two superfamilies have previously been analyzed by the SALSA method.^9^

In this work, two approaches, SALSA and GRASP‐Func, are used to predict the biochemical function of RPBB proteins of unknown function. Additionally, the second approach GRASP‐Func is applied to the 6‐HG and CAL/G superfamilies. First, the RPBB proteins of known function are used to generate Chemical Signatures for each of the functional families. Then the original SALSA method is applied, with alignments performed by conventional Cartesian‐coordinate‐based alignment programs on the entire protein structures, from which locally aligned sets of predicted active residues are generated. The 6‐HG and CAL/G superfamilies have been sorted previously with SALSA.^9^ We then present analysis of the three superfamilies with a new approach, wherein predicted sets of residues are expressed as graphs and local alignments are generated based on the graph representation. This new approach produces locally aligned signatures much faster and allows for more rapid, facile, larger‐scale functional classification of protein structures.

## Results and Discussion

### Chemical signatures based on Cartesian alignment of predicted residues using SALSA

The structures of proteins of known function in each superfamily were used to generate the Chemical Signatures for their respective superfamily and were chosen such that sequence homology between any two members within each family is as low as possible (Tables S3–S5, Supporting Information). For most families, at least two experimental structures are available within each family to establish the Chemical Signatures. For families with only one crystal structure available, homology models were generated using protein sequences in these functional families when available (Table S1, Supporting Information). The sequence identity matrix for the previously characterized protein structures in each superfamily was obtained using Clustal Omega^25^ and is given in Tables S3, S4, and S5. For each protein, the top 9% of POOL‐ranked residues were taken to be the predicted set of functional residues. Since the 6‐HG and CAL/G superfamilies have been analyzed previously,^9^ only the RPBB superfamily is analyzed by the SALSA method here.

Each superfamily is divided up into its respective functional families. Upon structural alignment of 31 selected RPBB proteins of known function (Table S2, Supporting Information), POOL‐predicted residues were found in 24 of the aligned spatial positions and are divided into nine functional families: indole‐3‐glycerol phosphate synthase (IGPS), tryptophan synthase (TrpA), phosphoribosyl anthranilate isomerase (PRAI), phosphoribosylformimino‐5‐aminoimidazole carboxamide ribotide isomerase (HisA), imidazole glycerol phosphate synthase (HisF), ribulose‐phosphate 3‐epimerase (RPE), orotidine 5′‐monophosphate decarboxylase (OMPDC), keto‐3‐gulonate‐phosphate decarboxylase (KGPDC), and hexulose phosphate synthase (HPS). Additionally, the structure of *E. coli* TrpC (PDB 1pii) in RPBB is bifunctional, where the N‐terminal domain (1–255) catalyzes the IGPS reaction and the C‐terminal domain (256–452) catalyzes the PRAI reaction.^26^ The alignment of the predicted residues for these 31 previously characterized proteins is shown in Table 1, in which each row represents a protein structure, with proteins of common biochemical function grouped together. The vertical columns represent spatially aligned positions, obtained from Cartesian‐based alignment of the complete structures. POOL‐predicted residues are shown in uppercase; aligned residues not predicted are in lowercase. The Chemical Signature residues are highlighted in yellow. Amino acids previously identified as important for catalysis, either from experimental evidence^27^, ^28^, ^29^, ^30^, ^31^, ^32^, ^33^, ^34^, ^35^, ^36^, ^37^, ^38^ or by sequence homology with an experimentally characterized protein,^39^ are shown in boldface. The normalized SALSA scores for the known members of this superfamily are given in Table S6, Supporting Information. Table I shows that each functional family within RPBB has a unique set of predicted residue types in aligned spatial positions; these local sets of structurally aligned, predicted residues that are common to a particular biochemical function constitute the Chemical Signature for that functional family, with a unique Chemical Signature for each functional family. For example, the Chemical Signature for the IGPS family consists of residues that are unique to the IGPS functional family, with the exception of Glu in column 16 (Table 1). In contrast, the KGPDC functional family consists of only one unique residue, Thr in column 21, and has a similar Chemical Signature to the HPS functional family. This is likely due to the promiscuity of members of the two families.^36^, ^37^

**Table 1:** SALSA Results for Functionally Characterized Members of the RPBB Superfamily

		Structure location of aligned residues																							
Group	PDB	1	2	3	4	5	6	7	8	9	10	11	12	13	14	15	16	17	18	19	20	21	22	23	24
IGPS	1pii:N	**E53**	**K55**	S85	l87	d89	–	–	y92	l112	**K114**	D115	F116	i118	m137	s139	**E163**	s165	G182	**N184**	R186	E214	**S215**	g236	s237
	1i4n	**E47**	**K49**	s79	L81	e83	–	–	y86	l106	**K108**	D109	F110	i112	i131	r133	**E157**	h159	g177	**N179**	R181	E209	**S210**	G231	T232
	2c3z	**E51**	**K53**	S81	l83	e85	–	–	y88	l108	**K110**	D111	F112	v114	I133	K135	**E159**	n161	G178	**N180**	R182	E210	**S211**	g233	s234
TrpA	1geq	Y10	t12	**E36**	G38	P40	**D47**	Q52	s54	v84	M86	t87	Y88	y93	v115	D116	l139	a141	**Y161**	v163	l165	G197	F198	G220	S221
	1qop	F22	t24	**E49**	g51	P53	**D60**	Q65	a67	G98	l100	m101	Y102	f107	a129	D130	i153	p155	**Y175**	l177	r179	g211	F212	G234	S235
	1xc4	F22	t24	E49	G51	p53	D60	q65	a67	G98	L100	M101	y102	f107	a129	D130	i153	p155	Y175	l177	r179	g211	F212	g213	S233
	1rd5	Y23	t25	E50	G52	P54	D61	Q66	s68	v98	l100	s101	Y102	m107	p125	D126	l149	t151	Y171	v173	v175	G207	F208	G209	G230
PRAI	1pii:C	C260	G261	G280	i282	v284	–	s287	R289	v308	v310	f311	R312	d315	H334	N336	a358	s360	v377	D379	f389	A405	G406	S428	a429
	1lbm	**C7**	G8	G27	v29	y31	–	s34	R36	v57	v59	f60	v61	e64	H83	E85	a103	g105	l124	**D126**	f139	S157	G158	s181	g182
HisA	1qo2	a6	D8	H48	v50	D51	l52	s53	–	q77	g79	g80	g81	r98	s102	s103	s125	D127	v162	T164	D169	A194	g195	v222	g223
	1vzw	a9	D11	H50	v52	D53	l54	d55	–	E79	s81	g82	g83	R100	g104	t105	g128	D130	v164	T166	D171	S196	g197	g222	k223
	2y85	A9	D11	H50	V52	D53	L54	D55	–	E79	S81	G82	g83	R100	g104	t105	G128	D130	V168	T170	D175	S200	g201	g226	k227
HisF	1thf	C9	**D11**	v48	l50	D51	i52	t53	–	t78	g80	g81	g82	K99	N103	t104	a128	**D130**	l169	t171	D176	S201	g202	a224	s225
	1h5y	C10	**D12**	a51	l53	D54	i55	t56	–	l81	g83	g84	g85	K102	n106	t107	a131	**D133**	l172	t174	D179	S204	g205	A227	s228
	1ox6	C243	**D245**	t295	l297	n298	i299	t300	–	t328	g330	g331	g332	K360	g364	t365	S402	**D404**	L467	N469	D474	S499	S500	A523	g524
RPE	1rpx	S16	l18	**H41**	**D43**	M45	–	p51	i53	D72	**H74**	l75	M76	d81	H98	E100	v124	l125	l145	M147	v149	**D185**	g186	g207	s208
	2fli	S9	l11	H34	D36	M38	–	p44	i46	D65	H67	l68	M69	e74	H91	E93	v115	i116	l136	M138	v140	D176	g177	g198	s199
	1h1y	S11	l13	**H36**	**D38**	M40	–	p46	l48	D67	**H69**	l70	M71	s76	H93	E95	s118	l119	l142	m144	v146	**D178**	g179	g200	s201
	1tqj	S10	l12	H35	D37	M39	–	p45	I47	D66	H68	l69	M70	e75	H92	E94	v118	l119	l139	M141	v143	D179	g180	g201	s202
	3ovp	S10	l12	H35	D37	m39	–	p45	I47	D68	H70	m71	m72	e77	H94	E96	a118	i119	l139	m141	v143	D175	g176	G197	s198
OMPDC	1dbt	a9	D11	K33	g35	M36	–	–	–	F58	**D60**	l61	**K62**	**D65**	H88	a90	v119	q121	V160	s162	–	P182	g183	g214	R215
	1dv7	A18	D20	K42	g44	y45	–	–	–	i68	D70	f71	K72	D75	H98	f100	l123	e125	v155	p157	–	P180	g181	g202	R203
	1dqw	s35	D37	K59	H61	v62	–	–	–	F89	D91	r92	K93	D96	H122	v124	l150	e152	i183	q185	–	P202	G203	G234	R235
	1l2u	a20	D22	K44	g46	k47	–	–	–	F69	**D71**	l72	**K73**	**D76**	H99	s101	v127	v129	v167	s169	–	P189	G190	g221	R222
	2za1	G21	D23	K102	H104	f105	–	–	–	I134	D136	m137	K138	D141	n165	Y167	l191	k193	V240	g242	–	P264	G265	g293	R294
	3qw3	G19	D21	K49	n51	a52	–	–	–	v80	D82	a83	K84	d87	s111	y113	l133	K135	v175	g177	–	P199	G200	s228	R229
	3l0k	S33	D35	K57	H59	v60	–	–	–	F86	D88	r89	K90	d93	H119	y121	i144	e146	i177	g179	–	P193	g194	G226	R227
KGPDC	1xbv	A9	D11	E33	G35	T36	I37	l38	C39	l60	D62	a63	**K64**	**D67**	I87	C88	E112	t114	**H136**	s138	r139	T169	G170	G191	R192
	3exr	A11	D13	E35	G37	t38	t39	c40	l41	v62	D64	t65	K66	D69	i89	c90	E117	Y119	H141	s143	r144	T174	G175	G196	R197
HPS	3ajx	A6	D8	E30	G32	T33	P34	l35	i36	F57	D59	m60	**K61**	D64	L84	g85	D109	I111	**H134**	g136	l137	A164	g165	G186	g187
	HPS1	A6	D8	E30	G32	T33	P34	v35	v36	l57	D59	l60	K61	d64	l84	g85	D109	i111	H134	g136	y137	a165	g166	G187	G188

Each row represents a protein structure, with proteins of common function grouped together. The vertical columns represent spatially aligned positions, obtained from Cartesian‐based alignment of the complete structures. POOL‐predicted residues are shown in uppercase; aligned residues not predicted are in lowercase. Previously reported catalytic residues are shown in **boldface**. The Chemical Signature residues are shaded in yellow.

In the 6‐HG superfamily, SALSA has previously characterized the proteins of known function into 13 functional families: 1,4‐α‐l‐glucan glucohydrolase (AGG), exo‐α‐1,6‐mannosidase (AMAN), endoglucanase/xylanase/chitosanase (EXC), cellulases (CELL), unsaturated glucuronyl hydrolase (UGH), α‐l‐rhamnosidase (ALR), 1,2‐α‐l‐fucosidase and α‐l‐galactosidase (ALF/ALG), trehalase (TRE), unsaturated rhamnogalacturonyl hydrolase (URH), α‐amylase (AMY), phosphorylase I (CDP), phosphorylase II (NGP), and *N*‐acylglucosamine‐2‐epimerase (NAE).^9^ Additionally, SALSA previously characterized the proteins of known function in the CAL/G superfamily into six functional families: xylanases (XYL), endoglucanases (ENDO), cellobiohydrolases (CBH), GH family 16 (GH16), lyases (ALY), and peptidases (PEP).^9^ For these two superfamilies, the normalized SALSA scores for the known members are given in Tables S8 and S10, Supporting Information.

### Application of SALSA to the SG members of the RPBB superfamily

The SG members of each superfamily were found from searches for proteins with a sequence or keyword match, or structural similarity to previously characterized proteins in each respective superfamily. These SG proteins, with the sources of their structures, are listed in the Table S12, Supporting Information. In the RPBB superfamily, the SG proteins are aligned with previously characterized proteins (Table 1), and the aligned, POOL‐predicted residues for the SG proteins are scored against the Chemical Signatures for the nine functional families.

The match score MS for SG protein j with the Chemical Signature CS for family k, calculated using scoring matrix **M**, is obtained as:

(1) $${\text{MS}}_{\text{jk}}=\;<{\text{CS}}_{k}\left|M\right|{\text{SG}}_{j}>$$

Normalized match scores S are calculated as:

(2) $$S_{\text{jk}}=\;<{\text{CS}}_{k}\left|M\right|{\text{SG}}_{j}>/<{\text{CS}}_{k}\left|M\right|{\text{CS}}_{k}>$$

so that a perfect match of aligned residues of the SG protein with those of the Chemical Signature for family k yields a score S of 1. For present purposes, the BLOSUM62^40^, ^41^ scoring matrix was used in Eqs. (1) and (2).

Table S7 (Supporting Information) shows the normalized match scores S for 44 SG proteins against the Chemical Signatures for the nine functional families in the RPBB superfamily. For each functional family, the number of aligned positions N in the Chemical Signature is given in the first row. In the next row, for functional families with more than two previously characterized proteins, the range of S values within the set of previously characterized members is given (Table S6, Supporting Information). Table S7 (Supporting Information) reveals that 41 of the 44 SG proteins have high scores with one functional family and substantially lower scores with the other eight functional families. In some instances, a protein exhibiting a strong match with one function and a moderate match with another function (i.e., putative hexulose‐6‐phosphate synthase SgbH from *Vibrio cholerae*, PDB 3ieb) may exhibit some promiscuity, as has been observed for previously characterized KGPDC and HPS enzymes.^36^, ^37^ The last two proteins shown in Table S7 (two putative *N*‐acetylmannosamine‐6‐phosphate 2‐epimerases, PDBs 1y0e and 1yxy) have scores below +0.10 with all nine functional families. These two proteins have similar structures to the members of the RPBB superfamily but have predicted function different from those of the RPBB proteins. For one of the superfamily members from *Saccharomyces cerevisiae*, originally annotated as a HisA/HisF protein (PDB 2agk), its highest score of +0.20 with the HisF family is too low to assign function and therefore it is unlikely to have any of the nine RPBB functions.

The highest match score is used to guide the SALSA functional assignment. Based on the ranges of normalized match scores obtained for the previously characterized proteins, a measure can be derived of the strength of the match to a given functional family. For each SG protein, if the highest normalized match score is greater than or equal to 0.90 or is within the range of scores obtained for the previously characterized proteins in a given functional family, then that highest score is labeled as a strong match (designated s). For normalized match scores less than the strong match threshold but greater than or equal to 0.70, the match strength is labeled moderate (m). Scores between 0.50 and 0.69 are labeled weak matches (w). Scores less than 0.50 are labeled “no match”. The top SALSA annotations for each SG protein, labeled (s), (m), or (w), are listed in Table S12, Supporting Information.

### Application of SALSA to the SG members of the 6‐HG and CAL/G superfamilies

Previously, several SG proteins in the 6‐HG and CAL/G superfamilies were analyzed using the SALSA method^9^; additional SG proteins are analyzed here. Aligning and scoring as described above, each SG protein was scored against each functional family in their respective superfamily. Table S9 (Supporting Information) shows the normalized match scores S for 11 SG proteins against the Chemical Signatures for 13 functional families in the 6‐HG superfamily. For each functional family, the number of aligned positions N in the Chemical Signature is given in the first row. In the next row, for functional families with more than two previously characterized proteins, the range of S values within the set of previously characterized members is given (Table S8, Supporting Information).

Table S9 (Supporting Information) reveals that fewer than half of the SG proteins can be sorted into a functional family reliably. Only uncharacterized protein BT_3781 from *Bacteroides thetaiotaomicron* (PDB 2p0v), uncharacterized protein BACOVA_03626 from *Bacteroides ovatus* (PDB 3on6), putative α‐rhamnosidase from *B. thetaiotaomicron* (PDB 3cih), and putative glycoside hydrolase protein BH0842 from *Bacillus halodurans* (PDB 2rdy) show strong matches with one functional family (AMAN, AMAN, ALR, and ALF/ALG, respectively). Interestingly, the two SG proteins showing a strong match with the AMAN functional family (PDB 2p0v and 3on6) also show weak matching with the AGG and TRE functional families, suggesting that these two SG proteins might display some promiscuity. In this superfamily, there are a few SG proteins that show weak matching with one functional family; putative alkaline invertase from *Nostoc sp*. (PDB 5goo) with AGG, two putative GH105 family proteins from *Klebsiella pneumoniae* (PDB 3pmm) and *Salmonella paratyphi* (PDB 3qwt) with UGH, and two putative *N*‐acetylglucosamine 2‐epimerases from *Salmonella typhimurium* (PDB 2afa) and *Xylella fastidiosa* (PDB 3gt5) with NAE. Two SG proteins, lin0763 protein from *Listeria innocua* (PDB 3k7x) and putative glycosyl hydrolase from *B. thetaiotaomicron* (PDB 4mu9) do not show significant normalized scores with any of the functional families. The top SALSA annotations for each SG protein, labeled (s), (m), or (w), are listed in Table S12, Supporting Information.

For the CAL/G superfamily, Table S11 (Supporting Information) shows the normalized match scores S for eight SG proteins against the Chemical Signatures for the six CAL/G functional families. Similar to Table S9 (Supporting Information), the number of aligned positions N in the Chemical Signature is given in the first row, followed by the range of S values within the set of previously characterized members (Table S10, Supporting Information). Table S11 (Supporting Information) reveals that one protein, putative GH16 family protein from *Mycobacterium smegmatis* (PDB 3rq0), has a score of +0.40. Normally, this would be considered “no match” according to our criteria; however, since the range of scores between the previously characterized members of the family is low (0.60–0.72) due to their different substrate specificities, we have assigned a weak functional annotation to this SG protein. Table S12 (Supporting Information) lists the SALSA results and shows that the other seven SG proteins have no match with any functional family we have analyzed. These SG proteins may be in functional families that lack structural coverage or are novel functional families.

### Function prediction with a graph theory approach (GRASP‐Func)

Here we introduce a computationally faster approach to sorting superfamilies according to biochemical function. For each protein structure in each superfamily, the set of highly‐ranked POOL residues is represented as a set of points in 3D space to form a graph representation, generated by Delaunay triangulation, of the active site. These graph representations can match rapidly one active site to another. The topological graph descriptors represent each predicted residue as a single point in space, using the coordinates of the α carbon atoms. This generates a set of tetrahedra, where the residues are represented by the vertices and the edges indicate that the two joined residues are neighbors. Delaunay triangulation has been used previously for protein structural alignment by common volume superposition^42^; here it is applied to identify similar spatially localized regions of structures.

**Figure 1:**
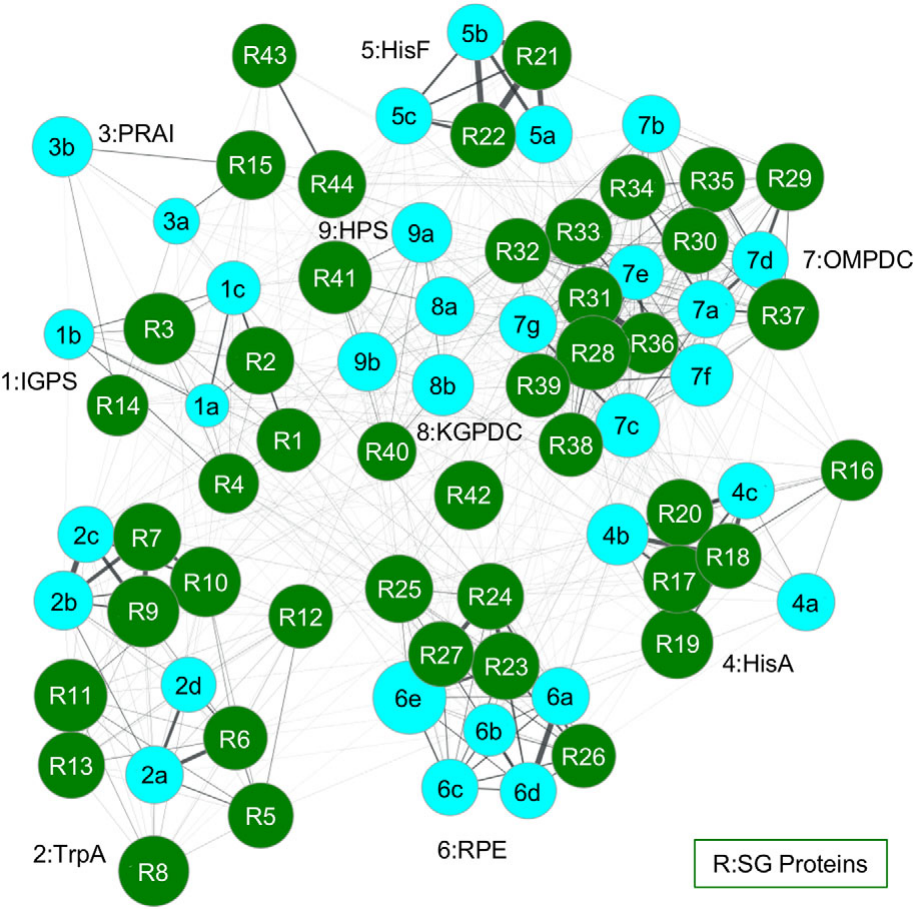
GRASP‐Func clustering of RPBB known function (light blue) and SG (dark green) proteins. Proteins are represented as nodes. The thickness of each edge shows the degree of similarity between the two connected proteins. PDB IDs for proteins of known function: 1pii:N, 1i4n, 2c3z (1a–c, respectively); 1geq, 1qop, 1xc4, 1rd5 (2a–d); 1pii:C, 1lbm (3a–b); 1qo2, 1vzw, 2y85 (4a–c); 1thf, 1h5y, 1ox6 (5a–c); 1rpx, 2fli, 1h1y, 1tqj, 3ovp (6a–e); 1dbt, 1dv7, 1dqw, 1l2u, 2za1, 3qw3, 3l0k (7a–g); 1xbv, 3exr (8a–b); 3ajx, HPS1 (9a–b). Each SG protein is numbered based on its Label in Table S12, Supporting Information.

**Figure 2:**
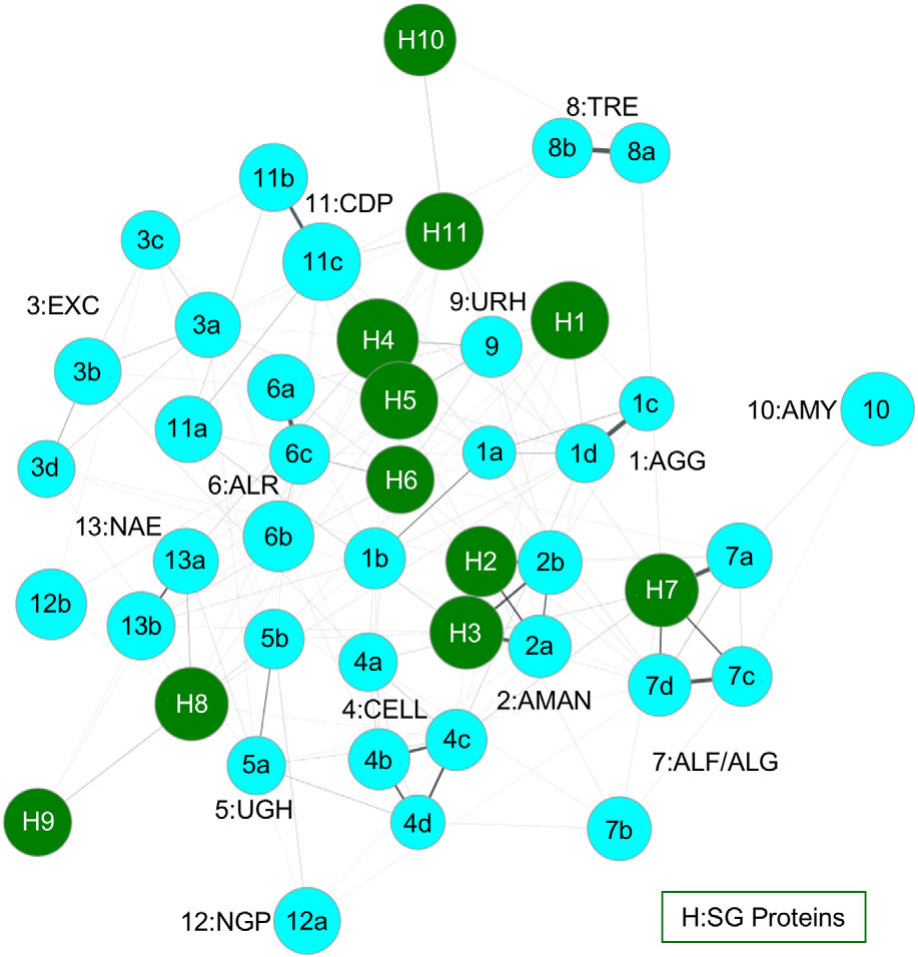
GRASP‐Func clustering of 6‐HG known function (light blue) and SG (dark green) proteins. Proteins are represented as nodes. The thickness of each edge shows the degree of similarity between the two connected proteins. PDB IDs for proteins of known function: 1gai, 1ayx, 1lf9, 1ug9 (1a–d); 3qt9, 3qsp (2a–b); 1cem, 1wu4, 1v5c, 1h12 (3a–d); 1clc, 1kfg, 1ksc, 1ia6 (4a–d); 2d5j, 2zzr (5a–b); 2okx, 3w5m, ALR1 (6a–c); 4ufc, 2eac, ALF1, ALF2 (7a–d); 2jf4, TRE1 (8a–b); 2d8l (9); 3ren (10); 1v7x, 2cqs, CDP1 (11a–c); 1h54, NGP1 (12a–b); 1fp3, 2gz6 (13a–b). Each SG protein is numbered based on its Label in Table S12, Supporting Information.

**Figure 3:**
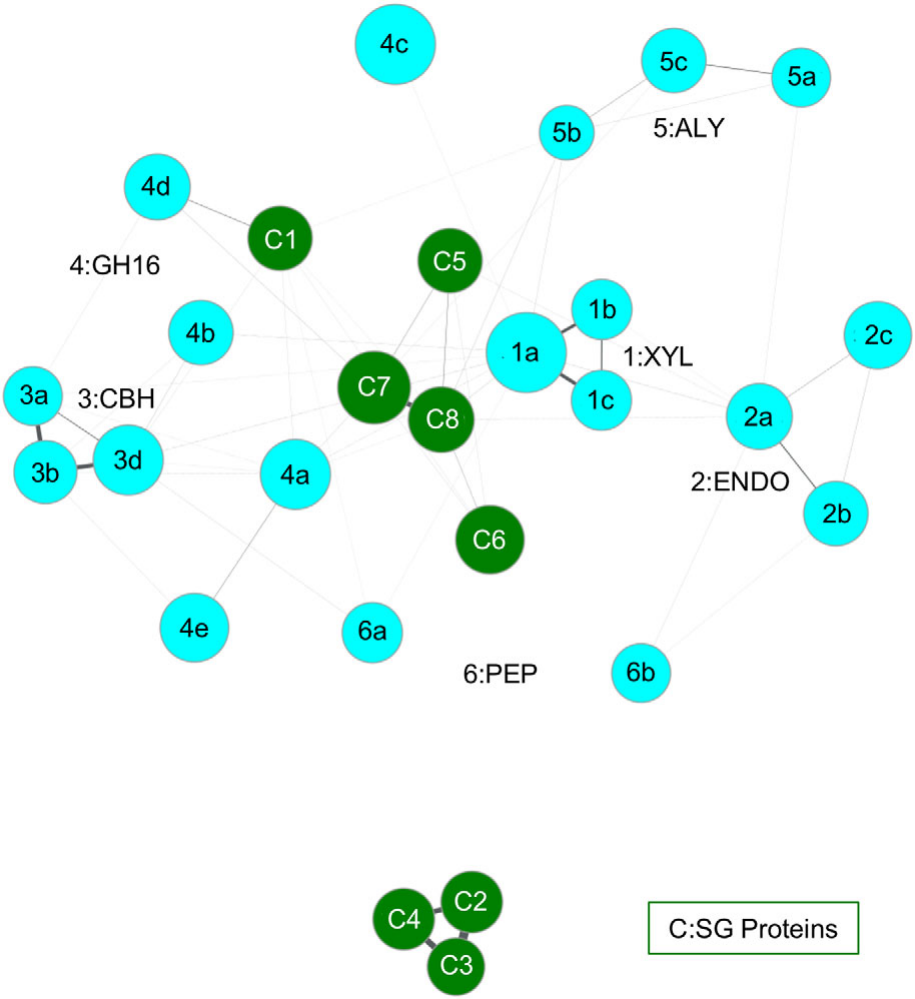
GRASP‐Func clustering of CAL/G known function (light blue) and SG (dark green) proteins. Proteins are represented as nodes. The thickness of each edge shows the degree of similarity between the two connected proteins. PDB IDs for proteins of known function: 1m4w, 1h4g, 1bcx (1a–c); 1uu4, 1h8v, 2nlr (2a–c); 1z3t, 1dy4, 2rfw (3a–c); 2ayh, 1dyp, 3ilf, 2vy0, 1mve (4a–e); 1uai, 1j1t, 1vav (5a–c); 2fir, 1y43 (6a–b). Each SG protein is numbered based on its label in Table S12, Supporting Information.

The sets of tetrahedra that contain POOL‐predicted residues for a pair of proteins are then compared using a pairwise matching algorithm, described in the Methods section. Sets of proteins with matched tetrahedra are then grouped together by this algorithm. Matches between sets of proteins of known function with a query protein of unknown function thus enable function prediction for the query protein. One of the main advantages of GRASP‐Func over SALSA is that GRASP‐Func does not rely on global structural alignments, which can be very time consuming and labor intensive. Additionally, when analyzing function similarity across folds, SALSA requires a manual alignment process^4^ while GRASP‐Func can analyze function without the need for global alignments. While SALSA makes function predictions using a table of spatially aligned, functionally important residues for protein structures within a superfamily (as illustrated in Table 1), GRASP‐Func uses similarity between sets of four‐membered graphs and generates a figure showing the proteins of similar function grouped together; individual proteins are represented as nodes and the thickness of each edge shows the degree of similarity between the two connected proteins (as illustrated in Figs. 1, 2, 3). GRASP‐Func was optimized with the RPBB superfamily; 6‐HG and CAL/G superfamilies were then used to test the method.

In the RPBB superfamily, the previously characterized proteins listed in Table S2 (Supporting Information) are sorted correctly into nine groups by GRASP‐Func (Fig. S3, Supporting Information). This correct classification into nine functional families is the same as the SALSA classification shown in Table 1. In the 6‐HG superfamily, the previously characterized proteins are sorted into 13 groups by GRASP‐Func (Fig. S4, Supporting Information). This functional classification is similar to the SALSA classification, with the exception of the Phosphorylase II family (Group 12). The maltose phosphorylase from *Lactobacillus brevis* (PDB 1h54) and the nigerose phosphorylase from *Clostridium phytofermentans* (homology model NGP1) do not show a correlation using this method. This is attributed to the homology model generated for nigerose phosphorylase, which was built from the maltose phosphorylase crystal structure (PDB 1h54) template but has a low model quality score^9^ (Table S1, Supporting Information). The model structure was analyzed by PROCHECK,^43^ and the results showed only 88.2% of the nonglycine/proline residues (605 residues) are in the most favored regions, 10.1% (69 residues) in additionally allowed regions, 1.2% (8 residues) in generously allowed regions, and 0.6% (4 residues) in disallowed regions. A good quality model is expected to show 90% or more of the nonglycine/proline residues in favored regions. The residues in the generously and disallowed regions are located distal from the active site and may disrupt the network within the protein structure. Similarly, the 19 previously characterized proteins in the CAL/G superfamily are sorted into six biochemical functional groups by GRASP‐Func (Fig. S5, Supporting Information), with the same classification as that of SALSA. The GH family 16 functional family (Group 4) shows some separation due to the different substrate specificities of the proteins of known function.

### Application of GRASP‐Func to SG proteins

Next, SG proteins listed in Table S12 (Supporting Information) were added to the GRASP‐Func analysis for each superfamily; functional assignments by SALSA and by GRASP‐Func are also listed in Table S12 (Supporting Information). In the RPBB superfamily, GRASP‐Func is able to assign the same function as SALSA to each SG protein (Fig. 1), only much faster, categorizing 44 SG proteins in 15 min; in this example GRASP‐Func has not sacrificed accuracy for speed. In comparison, the analysis of the proteins of known function with SALSA took ∼12 h, while the analysis of all proteins, known and SG, took several days.

The 6‐HG superfamily proteins were sorted by GRASP‐Func (Fig. 2), and the results show that for seven of the 11 SG proteins, GRASP‐Func is able to assign the same function as SALSA (Table S12, Supporting Information). The two putative GH105 family proteins from *K. pneumoniae* (PDB 3pmm, H4) and *S. paratyphi* (PDB 3qwt, H5) are assigned a weak (+0.51) UGH function by SALSA but are assigned a URH function by GRASP‐Func. Both families function by hydrolyzing their respective substrates and have a number of similar residues in their active sites.^9^ However, SALSA can only obtain a reliable Chemical Signature if the family has two or more protein structures and/or sequences of known function. In this case, the URH functional family has only one known representative. It is possible that SALSA assigned UGH function over URH function because a reliable Chemical Signature for URH is unavailable. In contrast, GRASP‐Func does not rely on the Chemical Signatures and global structural alignments and is able to provide functional annotations with only one known representative. Putative α‐l‐fucosidase from *Bacillus halodurans* (PDB 2rdy, H7 in Fig. 2) is predicted to be in the ALF/ALG functional family. Upon further analysis with individual members of the functional family, SALSA predicts galactosidase function. In GRASP‐Func, there is a strong match between this SG protein and the galactosidase function, as illustrated in Figure 2 by the darker edge connecting it to α‐l‐galactosidase from *Bacteroides ovatus* (PDB 4ufc, 7a in Fig. 2). Two SG proteins, putative GH76 family protein from *Listeria innocua serovar 6a* (PDB 3k7x, H10) and putative glycosylhydrolase from *Bacteroides thetaiotaomicron* (PDB 4mu9, H11) are unable to be annotated by either method. It is possible they are members of new functional families.

The CAL/G superfamily proteins were also sorted by GRASP‐Func (Fig. 3). In this instance, only one SG protein, putative GH family 16 from *Mycobacterium smegmatis* (PDB 3rq0, C1 in Fig. 3) is able to be assigned function by both SALSA and GRASP‐Func, in this case as having GH family 16 function (Table S12, Supporting Information). Specifically, Figure 3 shows that this protein likely has endo‐β‐1,3‐glucanase activity. While neither SALSA nor GRASP‐Func can assign function to the other seven SG proteins, GRASP‐Func shows that the three putative β‐xylosidase (PDBs 1y7b, 1yif, and 1yrz, C2−4 in Fig. 3, respectively) cluster together away from the other families and have a strong connection to each other as shown by the thick edges. Similarly, the two putative sugar hydrolases (PDBs 3h3l and 3nmb, C5 and C7 in Fig. 3, respectively) and the two putative glycosyl hydrolases (PDBs 3hbk and 3osd, C6 and C8 in Fig. 3, respectively) form a four‐membered, well‐connected cluster. These two clusters could represent new functional families in the superfamily.

The amount of time it takes to sort a set of proteins with GRASP‐Func varies, depending on the degree of similarity between pairs; sets with higher variability discard larger numbers of pairs early and therefore the sorting proceeds faster. In a typical run on an Intel Xeon E3–1220 v3 CPU running at 3.10 GHz, with 16 GB of RAM, it took 15 min of clock time to obtain 2240 results. This is at least several orders of magnitude faster than the full structural alignment employed in the original SALSA method, which can take hours to run depending on the size of the superfamily being analyzed. In addition, SALSA often requires manual adjustments, or unification of multiple, smaller alignments, to obtain the best local alignments, particularly for large sets of structures. GRASP‐Func also enables matching of functional types across folds; while this is possible in the original SALSA method,^9^ it is slow and labor intensive because manual alignments are required.

SALSA and GRASP‐Func both incorporate computed chemical properties from the POOL method to predict protein function from 3D structure. Both methods are based on structure similarity at the local site of biochemical activity and both have successfully sorted members of the three superfamilies into families according to predicted biochemical function. The graph representations of GRASP‐Func obviate global Cartesian alignments and therefore yield local‐structure‐based function assignments substantially faster and can be fully automated. Faster protein function annotation methods like GRASP‐Func will help correct function misannotations in databases and provide the scientific community with correct information. This will add a substantial amount of information to the already extensive amount of work done through SG efforts.

## Materials and Methods

### POOL predictions

POOL predictions were made as described by Somarowthu et al.^18^

### SALSA predictions based on Cartesian alignments

SALSA predictions were made as described by Wang *et al*.^15^ The top 9% of the residues in the POOL rankings were taken to be the predicted, functionally active residues that are marked in the structural alignments. When more than half of the proteins in a functional family have POOL‐predicted residues of common type in an aligned position, that residue becomes part of the Chemical Signature.

### GRASP‐Func Analysis

The protein structures were preprocessed to convert the coordinates into a set of tetrahedra and to identify the tetrahedra near the active site, based on the POOL rankings. To achieve this, first Delaunay triangulation was performed on the protein structure using Qhull.^44^ The vicinity of the active site is determined by the top 50 residues in the POOL rankings. All tetrahedra that contain a POOL‐predicted residue, or have a vertex connected to a POOL‐predicted residue, are collected for matching analysis. In a pair of proteins P_1_ and P_2_, the tetrahedra in the active site vicinity that have been identified in the preprocessing step are compared and seed pairs are sought. Seed pairs are ranked using POOL rank, residue similarity as measured by the BLOSUM62^40,^ 41 matrix, and lengths of the edges of the tetrahedra. If tetrahedron t_j,1_ in protein P_1_ and tetrahedron t_k,2_ in protein P_2_ have residues with high POOL rankings and chemical similarity, then the pair t_j,1_ and t_k,2_ is a seed pair. Then seed pairs of tetrahedra are compared according to the edge lengths, that is the distances between α carbon atoms. Additional features of a tetrahedron used in the matching algorithm are the volume, the sum of the lengths of the edges, and the relative orientation. The average volume for a tetrahedron in the RPBB superfamily is 14.4 Å^3^, so pairs of tetrahedra with a volume difference greater than 14.4 Å^3^ are rejected. The average sum of edge lengths is 9.6 Å, so pairs are rejected if total edge length difference exceeds 9.6 Å. Then the vertices, which represent the individual amino acids, are analyzed further. With the set of surviving pairs, the vertex pairs v_j,m,1_ in t_j,1_ from P_1_ and v_k,n,2_ in t_k,2_ from P_2_, where m and n are indices for the individual vertices in the tetrahedron, are further filtered according to the following sequential steps:

1. If v_j,m,1_ or v_k,n,2_ is among the top 11 POOL‐ranked residues in P_1_ and P_2_, respectively, and v_j,m,1_ is not chemically similar to v_k,n,2_, the pair is rejected.
2. If v_j,m,1_ or v_k,n,2_ is among the top 24 POOL‐ranked residues in its respective protein and the difference in POOL rank between v_j,m,1_ and v_k,n,2_ exceeds 24, the pair is rejected.
3. If v_j,m,1_ or v_k,n,2_ is among the top 10 POOL‐ranked residues in its respective protein and the difference in POOL rank between v_j,m,1_ and v_k,n,2_ exceeds 10, the pair is rejected.
4. If v_j,m,1_ or v_k,n,2_ is among the top three POOL‐ranked residues in its respective protein and the difference in POOL rank between v_j,m,1_ and v_k,n,2_ exceeds 3, the pair is rejected.

The final match of subgraphs for the two proteins includes matching residues and matching tetrahedra, using the best match scores based on POOL rank and chemical similarity. A link to the source code for the method can be found in the supplementary material.

## Supporting information

Supporting Information
