# Supplementary material for: Functional classification of protein structures by local structure matching in graph representation
Source: Protein Sci. 2018 Apr 27;27(6):1125–35. doi: 10.1002/pro.3416 (PMC5980557; doi:10.1002/pro.3416)
Supplement: Supplementary file 1 — Supporting Information [file PRO-27-1125-s001.docx]

**Functional classification of protein structures by local structure matching in graph representation**

Caitlyn L. Mills^¶^, Rohan Garg^¶^, Joslynn S. Lee^¶^, Liang Tian, Alexandru Suciu, Gene Cooperman, Penny J. Beuning, Mary Jo Ondrechen^*^

Northeastern University, Boston, MA 02115 USA

**Supplementary Material:**

**GRASP-Func** files as well as instructions are available at: https://github.com/graspfunc

**Table S1. Homology models generated of protein sequences of known function in the RPBB and 6-HG superfamilies.**

| **UniProt ID** | **Superfamily** | **Model Label** | **Target Protein Name** | **Target Protein Source** | **Template PDB ID** | **Overall Quality Z-score** | **Model Quality Comment** | **Ref** |
| --- | --- | --- | --- | --- | --- | --- | --- | --- |
| P42405 | RPBB | HPS1 | 3-Hexulose-6-phosphate synthase | *Bacillus subtilis* | 3ajx | -0.796 | Good | [1] |
| Q6RCI8 | 6-HG | ALR1 | α-l-Rhamnosidase | *Thermomicrobia bacterium* PRI-1686 | 2okx | -1.265 | Satisfactory | [2] |
| Q8L7W8 | 6-HG | ALF1 | 1,2-α-l-Fucosidase | *Arabidopsis thaliana* | 2eac | -2.294 | Poor | [3] |
| A8J657 | 6-HG | ALF2 | 1,2-α-l-Fucosidase | *Lilium longiflorum* | 2eac | -2.534 | Poor | [4] |
| A8J4S9 | 6-HG | TRE1 | Trehalase | *Apis mellifera* | 2jf4 | -1.113 | Satisfactory | [5, 6] |
| P77846 | 6-HG | CDP1 | Cellodextrin Phosphorylase | *Clostridium stercorarium* | 1v7x, 2cqs | -1.222 | Satisfactory | [7] |
| A9KT32 | 6-HG | NGP1 | Nigerose Phosphorylase | *Clostridium phytofermentans* | 1h54 | -1.736 | Satisfactory | [8] |

**Table S2. Previously characterized protein structures from the RPBB superfamily, grouped according to functional family.**

| **Family** | **Pfam** | **E.C.** | **PDB** | **Species** | **Resolution (Å)** | **Ref** |
| --- | --- | --- | --- | --- | --- | --- |
| Indole-3-glycerol phosphate synthase  **(IGPS)** | PF00218 | 4.1.1.48 | 1pii(N)* | *Escherichia coli* | 2.00 | [9] |
|  |  |  | 1i4n | *Thermotoga maritima* | 2.50 | [10] |
|  |  |  | 2c3z | *Sulfolobus solfataricus* | 2.80 | [11] |
| Tryptophan synthase  **(TrpA)** | PF00290 | 4.2.1.20 | 1geq | *Pyrococcus furiosus* | 2.00 | [12] |
|  |  |  | 1qop | *Salmonella enterica* | 1.40 | [13] |
|  |  |  | 1xc4 | *Escherichia coli* | 2.80 | [14] |
|  |  |  | 1rd5 | *Zea mays* | 2.02 | [15] |
| Phosphoribosyl anthranilate isomerases  **(PRAI)** | PF00697 | 5.3.1.24 | 1pii(C)* | *Escherichia coli* | 2.00 | [9] |
|  |  |  | 1lbm | *Thermotoga maritima* | 2.80 | [16] |
| Phosphoribosylformimino-5-aminoimidazole carboxamide ribotide isomerase  **(HisA)** | PF00977 | 5.3.1.16 | 1qo2 | *Thermotoga maritima* | 1.85 | [17] |
|  |  |  | 1vzw | *Streptomyces coelicolor* | 1.80 | [18] |
|  |  |  | 2y85 | *Mycobacterium tuberculosis* | 2.40 | [19] |
| Imidazoleglycerolphosphate synthase  **(HisF)** | PF00977 | 4.1.3 | 1thf | *Thermotoga maritima* | 1.45 | [17] |
|  |  |  | 1h5y | *Pyrobaculum aerophilum* | 2.00 | [20] |
|  |  |  | 1ox6 | *Saccharomyces cerevisiae* | 2.40 | [21] |
| Ribulose-phosphate 3-epimerase  **(RPE)** | PF00834 | 5.1.3.1 | 1rpx | *Solanum tuberosum* | 2.30 | [22] |
|  |  |  | 2fli | *Streptococcus pyogenes* | 1.80 | [23] |
|  |  |  | 1h1y | *Oryza sativa* | 1.87 | [24] |
|  |  |  | 1tqj | *Synechocystis sp.* | 1.60 | [25] |
|  |  |  | 3ovp | *Homo sapiens* | 1.70 | [26] |
| Orotidine 5ʹ-monophosphate decarboxylase  **(OMPDC)** | PF00215 | 4.1.1.23 | 1dbt | *Bacillus subtilis* | 2.40 | [27] |
|  |  |  | 1dv7 | *Methanothermobacter thermautotrophicus* | 1.80 | [28] |
|  |  |  | 1dqw | *Saccharomyces cerevisiae* | 2.10 | [29] |
|  |  |  | 1l2u | *Escherichia coli* | 2.50 | [30] |
|  |  |  | 2za1 | *Plasmodium falciparum* | 2.65 | [31] |
|  |  |  | 3qw3 | *Leishmania infantum* | 1.70 | [32] |
|  |  |  | 3l0k | *Homo sapiens* | 1.34 | [33] |
| Keto-3-gulonate-phosphate decarboxylase  **(KGPDC)** | PF00215 | 4.1.1.85 | 1xbv | *Escherichia coli* | 1.66 | [34] |
|  |  |  | 3exr | *Streptococcus mutans* | 1.70 | [35] |
| Hexulose phosphate synthase  **(HPS)** | PF00215 | 4.1.2.43 | 3ajx | *Mycobacterium gastri* | 1.60 | [36] |
|  |  |  | HPS1 | *Bacillus subtilis* | HM | - |

*This protein is bifunctional, where the N-terminal domain (1-255) catalyzes the IGPS reaction and the C-terminal domain (256-452) catalyzes the PRAI reaction [9].

**Table S3. Sequence identity matrix of the 31 proteins of known function in the RPBB superfamily using the multiple sequence alignment program Clustal Omega.**

| PDB ID | 1pii:N | 1i4n | 2c3z | 1geq | 1qop | 1xc4 | 1rd5 | 1pii:C | 1lbm | 1qo2 | 1vzw | 2y85 | 1thf | 1h5y | 1ox6 | 1rpx | 2fli | 1h1y | 1tqj | 3ovp | 1dbt | 1dv7 | 1dqw | 1l2u | 2za1 | 3qw3 | 3l0k | 1xbv | 3exr | 3ajx | HPS1 |
| --- | --- | --- | --- | --- | --- | --- | --- | --- | --- | --- | --- | --- | --- | --- | --- | --- | --- | --- | --- | --- | --- | --- | --- | --- | --- | --- | --- | --- | --- | --- | --- |
| 1pii:N | 100 | 31.3 | 34.8 | 13.3 | 12.5 | 13.8 | 13.3 | 14.7 | 14.4 | 14.6 | 14.0 | 14.5 | 12.7 | 16.6 | 17.0 | 13.5 | 12.8 | 12.1 | 11.0 | 13.2 | 8.6 | 10.0 | 8.9 | 8.7 | 10.3 | 11.3 | 12.6 | 9.7 | 8.3 | 15.2 | 10.8 |
| 1i4n | 31.3 | 100 | 33.9 | 16.2 | 16.0 | 16.0 | 13.5 | 17.0 | 14.7 | 13.2 | 14.5 | 14.4 | 14.6 | 17.2 | 15.1 | 11.3 | 12.0 | 14.2 | 16.3 | 12.9 | 13.6 | 21.3 | 8.0 | 10.5 | 9.1 | 10.5 | 13.6 | 13.7 | 14.4 | 13.7 | 16.4 |
| 2c3z | 34.8 | 33.9 | 100 | 14.4 | 14.2 | 14.9 | 16.4 | 11.1 | 16.6 | 19.4 | 14.4 | 13.0 | 16.1 | 15.9 | 17.2 | 18.2 | 14.4 | 17.1 | 17.0 | 18.1 | 11.4 | 14.7 | 6.9 | 10.7 | 10.4 | 10.6 | 11.4 | 14.1 | 14.8 | 7.6 | 12.9 |
| 1geq | 13.3 | 16.2 | 14.4 | 100 | 32.3 | 33.5 | 39.1 | 14.6 | 15.5 | 11.3 | 16.9 | 17.8 | 12.7 | 16.2 | 11.3 | 14.7 | 17.4 | 12.7 | 20.2 | 16.3 | 15.3 | 16.0 | 11.2 | 11.8 | 11.6 | 9.9 | 10.7 | 10.4 | 9.8 | 15.2 | 15.1 |
| 1qop | 12.5 | 16.0 | 14.2 | 32.3 | 100 | 85.5 | 31.8 | 13.7 | 11.5 | 16.0 | 16.5 | 17.5 | 11.9 | 13.5 | 17.8 | 14.6 | 11.8 | 12.9 | 15.0 | 12.2 | 14.8 | 13.9 | 10.2 | 13.8 | 9.5 | 12.0 | 15.7 | 12.6 | 15.4 | 16.4 | 10.9 |
| 1xc4 | 13.8 | 16.0 | 14.9 | 33.5 | 85.5 | 100 | 30.7 | 14.5 | 7.7 | 17.2 | 17.1 | 17.5 | 12.4 | 13.5 | 15.9 | 13.6 | 13.3 | 13.9 | 15.5 | 13.2 | 14.3 | 14.4 | 10.2 | 14.8 | 9.5 | 10.6 | 15.3 | 11.6 | 14.4 | 16.4 | 13.5 |
| 1rd5 | 13.3 | 13.5 | 16.4 | 39.1 | 31.8 | 30.7 | 100 | 15.4 | 13.2 | 17.0 | 15.7 | 14.2 | 12.2 | 15.6 | 17.6 | 14.9 | 13.1 | 15.2 | 17.3 | 15.4 | 12.2 | 20.2 | 13.1 | 14.1 | 10.0 | 9.3 | 14.2 | 12.7 | 12.6 | 16.6 | 15.8 |
| 1pii:C | 14.7 | 17.0 | 11.1 | 14.6 | 13.7 | 14.5 | 15.4 | 100 | 31.6 | 16.5 | 13.7 | 17.1 | 12.5 | 13.7 | 14.9 | 12.4 | 10.7 | 6.6 | 14.3 | 7.1 | 7.0 | 9.8 | 6.9 | 8.7 | 11.9 | 10.1 | 10.5 | 9.8 | 6.5 | 12.6 | 8.3 |
| 1lbm | 14.4 | 14.7 | 16.6 | 15.5 | 11.5 | 7.7 | 13.2 | 31.6 | 100 | 12.7 | 10.7 | 11.4 | 12.4 | 12.2 | 12.6 | 11.8 | 7.6 | 6.8 | 12.9 | 7.4 | 5.4 | 12.5 | 5.4 | 4.7 | 9.6 | 13.6 | 6.6 | 7.5 | 7.5 | 6.0 | 7.7 |
| 1qo2 | 14.6 | 13.2 | 19.4 | 11.3 | 16.0 | 17.2 | 17.0 | 16.5 | 12.7 | 100 | 27.0 | 25.7 | 20.1 | 24.3 | 18.9 | 12.2 | 6.2 | 11.2 | 10.9 | 7.5 | 13.4 | 10.7 | 9.2 | 9.2 | 10.5 | 13.8 | 10.0 | 10.6 | 13.0 | 9.0 | 14.1 |
| 1vzw | 14.0 | 14.5 | 14.4 | 16.9 | 16.5 | 17.1 | 15.7 | 13.7 | 10.7 | 27.0 | 100 | 68.3 | 23.9 | 25.0 | 19.6 | 12.2 | 11.8 | 14.1 | 13.2 | 12.8 | 13.3 | 11.7 | 11.8 | 15.1 | 9.1 | 15.7 | 12.6 | 13.7 | 17.2 | 17.5 | 16.1 |
| 2y85 | 14.5 | 14.4 | 13.0 | 17.8 | 17.5 | 17.5 | 14.2 | 17.1 | 11.4 | 25.7 | 68.3 | 100 | 23.2 | 28.1 | 21.3 | 13.5 | 11.8 | 13.1 | 14.0 | 14.9 | 16.9 | 13.1 | 11.5 | 17.0 | 11.5 | 16.0 | 10.4 | 15.5 | 18.5 | 16.9 | 14.2 |
| 1thf | 12.7 | 14.6 | 16.1 | 12.7 | 11.9 | 12.4 | 12.2 | 12.5 | 12.4 | 20.1 | 23.9 | 23.2 | 100 | 54.4 | 45.1 | 14.5 | 11.0 | 15.2 | 14.7 | 12.4 | 13.1 | 12.1 | 11.4 | 11.9 | 11.3 | 13.6 | 14.7 | 11.0 | 14.6 | 14.7 | 12.1 |
| 1h5y | 16.6 | 17.2 | 15.9 | 16.2 | 13.5 | 13.5 | 15.6 | 13.7 | 12.2 | 24.3 | 25.0 | 28.1 | 54.4 | 100 | 43.1 | 19.2 | 13.5 | 17.0 | 15.3 | 12.4 | 13.7 | 14.5 | 10.2 | 8.9 | 12.7 | 14.7 | 12.3 | 14.7 | 10.9 | 19.2 | 14.7 |
| 1ox6 | 17.0 | 15.1 | 17.2 | 11.3 | 17.8 | 15.9 | 17.6 | 14.9 | 12.6 | 18.9 | 19.6 | 21.3 | 45.1 | 43.1 | 100 | 13.2 | 10.4 | 12.4 | 13.7 | 11.6 | 11.1 | 10.9 | 12.0 | 10.7 | 9.9 | 12.8 | 16.2 | 12.2 | 15.2 | 14.9 | 12.5 |
| 1rpx | 13.5 | 11.3 | 18.2 | 14.7 | 14.6 | 13.6 | 14.9 | 12.4 | 11.8 | 12.2 | 12.2 | 13.5 | 14.5 | 19.2 | 13.2 | 100 | 46.4 | 40.7 | 67.9 | 38.1 | 16.8 | 16.3 | 11.8 | 16.5 | 15.8 | 13.4 | 14.1 | 14.3 | 17.2 | 17.6 | 17.3 |
| 2fli | 12.8 | 12.0 | 14.4 | 17.4 | 11.8 | 13.3 | 13.1 | 10.7 | 7.6 | 6.2 | 11.8 | 11.8 | 11.0 | 13.5 | 10.4 | 46.4 | 100 | 47.9 | 45.0 | 43.9 | 18.5 | 18.9 | 14.4 | 19.1 | 15.6 | 16.9 | 14.3 | 16.1 | 18.6 | 22.6 | 17.6 |
| 1h1y | 12.1 | 14.2 | 17.1 | 12.7 | 12.9 | 13.9 | 15.2 | 6.6 | 6.8 | 11.2 | 14.1 | 13.1 | 15.2 | 17.0 | 12.4 | 40.7 | 47.9 | 100 | 41.3 | 52.3 | 16.2 | 17.8 | 12.8 | 15.3 | 13.5 | 16.1 | 12.7 | 14.0 | 17.8 | 20.9 | 12.9 |
| 1tqj | 11.0 | 16.3 | 17.0 | 20.2 | 15.0 | 15.5 | 17.3 | 14.3 | 12.9 | 10.9 | 13.2 | 14.0 | 14.7 | 15.3 | 13.7 | 67.9 | 45.0 | 41.3 | 100 | 37.5 | 18.6 | 21.7 | 12.3 | 16.4 | 13.4 | 13.0 | 14.6 | 14.7 | 20.1 | 17.6 | 18.9 |
| 3ovp | 13.2 | 12.9 | 18.1 | 16.3 | 12.2 | 13.2 | 15.4 | 7.1 | 7.4 | 7.5 | 12.8 | 14.9 | 12.4 | 12.4 | 11.6 | 38.1 | 43.9 | 52.3 | 37.5 | 100 | 16.8 | 16.6 | 10.9 | 16.0 | 12.1 | 13.2 | 12.3 | 12.6 | 19.5 | 18.5 | 13.5 |
| 1dbt | 8.6 | 13.2 | 11.4 | 15.3 | 14.8 | 14.3 | 12.2 | 7.0 | 5.4 | 13.3 | 13.3 | 16.9 | 13.1 | 12.7 | 11.1 | 16.8 | 18.5 | 16.2 | 18.6 | 16.8 | 100 | 19.9 | 21.5 | 40.2 | 18.8 | 16.3 | 22.1 | 19.1 | 17.4 | 18.9 | 19.6 |
| 1dv7 | 10.0 | 21.3 | 14.7 | 16.0 | 13.9 | 14.4 | 20.2 | 9.8 | 12.5 | 10.7 | 11.7 | 13.1 | 12.1 | 14.5 | 10.9 | 16.3 | 18.9 | 17.8 | 21.7 | 16.6 | 19.9 | 100 | 17.3 | 21.8 | 23.5 | 23.6 | 21.7 | 25.5 | 23.8 | 28.6 | 28.1 |
| 1dqw | 8.9 | 8.0 | 6.9 | 11.2 | 10.2 | 10.2 | 13.1 | 6.9 | 5.4 | 9.2 | 11.8 | 11.5 | 11.4 | 10.2 | 12.0 | 11.8 | 14.4 | 12.8 | 12.3 | 10.9 | 21.5 | 17.3 | 100 | 19.1 | 15.1 | 18.2 | 49.4 | 13.6 | 16.2 | 14.1 | 15.5 |
| 1l2u | 8.7 | 10.5 | 10.7 | 11.9 | 13.8 | 14.8 | 14.1 | 8.7 | 4.7 | 9.2 | 15.1 | 17.0 | 11.9 | 8.9 | 10.7 | 16.5 | 19.1 | 15.3 | 16.4 | 16.0 | 40.2 | 21.8 | 19.1 | 100 | 15.6 | 13.4 | 18.6 | 17.4 | 21.0 | 17.1 | 16.3 |
| 2za1 | 10.3 | 9.1 | 10.4 | 11.6 | 9.5 | 9.5 | 10.0 | 11.9 | 9.6 | 10.5 | 9.1 | 11.5 | 11.3 | 12.7 | 9.9 | 15.8 | 15.6 | 13.5 | 13.4 | 12.1 | 18.8 | 23.5 | 15.1 | 15.6 | 100 | 32.6 | 16.4 | 16.7 | 17.4 | 16.8 | 17.6 |
| 3qw3 | 11.3 | 10.5 | 10.6 | 9.9 | 12.0 | 10.6 | 9.3 | 10.1 | 13.6 | 13.8 | 15.7 | 16.0 | 13.6 | 14.7 | 12.8 | 13.4 | 16.9 | 16.1 | 13.0 | 13.2 | 16.3 | 23.6 | 18.2 | 13.4 | 32.6 | 100 | 20.5 | 15.6 | 15.3 | 21.4 | 16.6 |
| 3l0k | 12.6 | 13.6 | 11.4 | 10.7 | 15.7 | 15.3 | 14.2 | 10.5 | 6.6 | 10.0 | 12.6 | 10.4 | 14.7 | 12.3 | 16.2 | 14.1 | 14.3 | 12.7 | 14.6 | 12.3 | 22.1 | 21.7 | 49.4 | 18.6 | 15.4 | 20.5 | 100 | 14.0 | 16.2 | 17.7 | 18.5 |
| 1xbv | 9.7 | 13.7 | 14.1 | 10.4 | 12.6 | 11.6 | 12.7 | 9.8 | 7.5 | 10.6 | 13.7 | 15.5 | 11.0 | 14.7 | 13.2 | 14.3 | 16.1 | 14.7 | 14.7 | 12.6 | 19.1 | 25.5 | 13.6 | 17.4 | 16.7 | 15.6 | 14.0 | 100 | 46.8 | 35.0 | 32.1 |
| 3exr | 8.3 | 14.4 | 14.8 | 9.8 | 15.4 | 14.4 | 12.6 | 6.5 | 7.5 | 13.0 | 17.2 | 18.5 | 14.6 | 10.9 | 15.2 | 17.2 | 18.6 | 17.8 | 20.1 | 19.5 | 17.4 | 23.8 | 16.2 | 21.0 | 17.4 | 15.3 | 16.2 | 46.8 | 100 | 29.1 | 27.8 |
| 3ajx | 15.2 | 13.7 | 7.6 | 15.2 | 16.4 | 16.4 | 16.6 | 12.6 | 6.0 | 9.0 | 17.5 | 16.9 | 14.7 | 19.2 | 14.9 | 17.6 | 22.6 | 20.9 | 17.6 | 18.5 | 18.9 | 28.6 | 14.1 | 17.1 | 16.8 | 21.4 | 17.7 | 35.0 | 29.1 | 100 | 39.1 |
| HPS1 | 10.8 | 16.4 | 12.9 | 15.1 | 10.9 | 13.5 | 15.8 | 8.3 | 7.7 | 14.1 | 16.1 | 14.2 | 12.1 | 14.7 | 12.5 | 17.3 | 17.6 | 12.9 | 18.9 | 13.5 | 19.6 | 28.1 | 15.5 | 16.3 | 17.6 | 16.6 | 18.5 | 32.1 | 27.8 | 39.1 | 100 |

The black outlined regions highlight the proteins within a subclass. A sequence identity greater than 35% is colored orange and as the percentage increases, it become red (>60%). Lower sequence identity is indicated by the colors yellow (>20%) and green (>10%).

**Table S4. Sequence identity matrix of the 34 proteins of known function in the 6-HG superfamily using the multiple sequence alignment program Clustal Omega.**

| PDB ID | 1gai | 1ayx | 1lf9 | 1ug9 | 3qt9 | 3QSP | 1cem | 1wu4 | 1v5c | 1h12 | 1clc | 1kfg | 1ksc | 1ia6 | 2d5j | 2zzr | 2okx | 3w5m | ALR1 | 4ufc | 2eac | ALF1 | ALF2 | 2jf4 | TRE1 | 2d8l | 3ren | 1v7x | 2cqs | CDP1 | 1h54 | NGP1 | 1fp3 | 2gz6 |
| --- | --- | --- | --- | --- | --- | --- | --- | --- | --- | --- | --- | --- | --- | --- | --- | --- | --- | --- | --- | --- | --- | --- | --- | --- | --- | --- | --- | --- | --- | --- | --- | --- | --- | --- |
| 1gai | 100 | 35.3 | 14.8 | 17.7 | 6.9 | 10.0 | 10.5 | 12.8 | 9.0 | 11.4 | 8.3 | 15.1 | 12.4 | 11.6 | 7.9 | 9.9 | 14.0 | 10.9 | 13.6 | 13.9 | 20.7 | 14.8 | 16.7 | 12.9 | 14.9 | 14.7 | 12.0 | 13.0 | 13.8 | 9.8 | 7.7 | 8.7 | 7.7 | 8.4 |
| 1ayx | 35.3 | 100 | 14.7 | 13.5 | 8.5 | 6.1 | 9.2 | 12.4 | 12.9 | 11.6 | 7.4 | 14.0 | 15.0 | 14.3 | 8.3 | 10.1 | 12.2 | 10.0 | 11.4 | 12.7 | 18.7 | 15.1 | 16.9 | 10.9 | 11.5 | 12.4 | 11.7 | 11.1 | 10.6 | 9.9 | 10.8 | 12.1 | 4.1 | 7.6 |
| 1lf9 | 14.8 | 14.7 | 100 | 39.5 | 11.0 | 7.5 | 10.9 | 7.8 | 12.3 | 9.3 | 10.3 | 12.1 | 13.3 | 11.2 | 10.7 | 10.7 | 11.7 | 12.9 | 12.9 | 13.8 | 15.8 | 14.2 | 13.0 | 11.2 | 12.3 | 11.1 | 12.1 | 10.0 | 11.7 | 11.5 | 10.1 | 8.0 | 7.2 | 6.7 |
| 1ug9 | 17.7 | 13.5 | 39.5 | 100 | 13.5 | 12.4 | 12.5 | 7.7 | 13.5 | 10.2 | 11.7 | 13.0 | 13.2 | 12.1 | 10.7 | 9.8 | 12.8 | 14.7 | 13.2 | 15.4 | 18.2 | 17.0 | 16.6 | 13.1 | 13.0 | 11.8 | 10.2 | 11.8 | 13.6 | 9.7 | 8.9 | 6.8 | 7.3 | 7.0 |
| 3qt9 | 6.9 | 8.5 | 11.0 | 13.5 | 100 | 45.8 | 8.8 | 8.7 | 7.1 | 8.3 | 10.3 | 5.9 | 4.0 | 7.1 | 10.2 | 10.6 | 11.2 | 8.3 | 10.8 | 7.8 | 7.7 | 4.9 | 8.4 | 4.7 | 8.8 | 8.9 | 13.7 | 13.2 | 15.9 | 16.6 | 8.3 | 11.8 | 13.1 | 5.6 |
| 3qsp | 10.0 | 6.1 | 7.5 | 12.4 | 45.8 | 100 | 10.1 | 5.9 | 6.1 | 9.3 | 8.9 | 5.9 | 5.7 | 10.6 | 10.1 | 12.4 | 11.9 | 6.9 | 11.1 | 10.5 | 9.0 | 11.1 | 7.3 | 6.7 | 16.0 | 8.9 | 10.5 | 14.6 | 15.7 | 17.2 | 11.0 | 9.9 | 9.4 | 6.5 |
| 1cem | 10.5 | 9.2 | 10.9 | 12.5 | 8.8 | 10.1 | 100 | 22.4 | 26.9 | 24.0 | 22.7 | 12.9 | 16.1 | 18.6 | 6.2 | 9.4 | 14.0 | 12.8 | 14.0 | 9.2 | 12.5 | 8.5 | 7.7 | 10.3 | 11.8 | 7.2 | 21.0 | 8.9 | 13.7 | 9.7 | 7.6 | 9.6 | 5.1 | 3.1 |
| 1wu4 | 12.8 | 12.4 | 7.8 | 7.7 | 8.7 | 5.9 | 22.4 | 100 | 17.1 | 34.5 | 9.6 | 15.2 | 13.4 | 12.5 | 6.5 | 6.3 | 9.3 | 14.3 | 11.3 | 10.0 | 10.7 | 11.7 | 11.4 | 8.0 | 8.2 | 8.2 | 18.4 | 9.8 | 11.0 | 8.1 | 9.0 | 10.1 | 7.9 | 8.0 |
| 1v5c | 9.0 | 12.9 | 12.3 | 13.5 | 7.1 | 6.1 | 26.9 | 17.1 | 100 | 20.9 | 16.9 | 19.7 | 18.3 | 18.2 | 9.6 | 11.1 | 10.8 | 12.4 | 8.9 | 11.4 | 14.2 | 8.3 | 11.9 | 9.5 | 11.3 | 11.5 | 19.8 | 4.3 | 5.5 | 7.2 | 9.5 | 8.8 | 3.0 | 6.1 |
| 1h12 | 11.4 | 11.6 | 9.3 | 10.2 | 8.3 | 9.3 | 24.0 | 34.5 | 20.9 | 100 | 11.7 | 14.5 | 13.8 | 16.5 | 6.1 | 10.1 | 11.2 | 14.4 | 8.6 | 9.7 | 12.5 | 9.2 | 10.7 | 9.2 | 10.1 | 8.7 | 18.2 | 8.3 | 7.1 | 9.8 | 8.3 | 6.6 | 5.0 | 5.1 |
| 1clc | 8.3 | 7.4 | 10.3 | 11.7 | 10.3 | 8.9 | 22.7 | 9.6 | 16.9 | 11.7 | 100 | 25.7 | 23.8 | 26.0 | 6.0 | 9.8 | 13.2 | 17.2 | 12.6 | 10.8 | 12.7 | 11.0 | 8.3 | 9.0 | 11.1 | 7.0 | 17.2 | 8.3 | 13.5 | 9.8 | 13.2 | 7.7 | 9.1 | 10.6 |
| 1kfg | 15.1 | 14.0 | 12.1 | 13.0 | 5.9 | 5.9 | 12.9 | 15.2 | 19.7 | 14.5 | 25.7 | 100 | 44.7 | 41.3 | 9.2 | 11.0 | 11.1 | 16.4 | 11.5 | 8.9 | 12.2 | 8.1 | 10.1 | 11.6 | 14.1 | 4.0 | 17.4 | 9.2 | 9.5 | 8.9 | 11.1 | 8.0 | 9.7 | 9.0 |
| 1ksc | 12.4 | 15.0 | 13.3 | 13.2 | 4.0 | 5.7 | 16.1 | 13.4 | 18.3 | 13.8 | 23.8 | 44.7 | 100 | 37.7 | 8.9 | 9.6 | 13.5 | 14.6 | 16.7 | 13.9 | 12.8 | 10.2 | 10.5 | 9.4 | 11.7 | 8.8 | 13.3 | 11.9 | 8.1 | 7.0 | 9.6 | 7.2 | 8.0 | 9.5 |
| 1ia6 | 11.6 | 14.3 | 11.2 | 12.1 | 7.1 | 10.6 | 18.6 | 12.5 | 18.2 | 16.5 | 26.0 | 41.3 | 37.7 | 100 | 7.5 | 15.3 | 13.1 | 14.0 | 11.2 | 9.2 | 10.6 | 11.5 | 10.1 | 7.8 | 10.2 | 4.1 | 14.5 | 13.0 | 12.0 | 8.6 | 11.7 | 10.0 | 6.5 | 8.8 |
| 2d5j | 7.9 | 8.3 | 10.7 | 10.7 | 10.2 | 10.1 | 6.2 | 6.5 | 9.6 | 6.1 | 6.0 | 9.2 | 8.9 | 7.5 | 100 | 42.3 | 19.5 | 10.8 | 17.3 | 9.7 | 9.2 | 15.0 | 9.9 | 15.7 | 11.2 | 7.3 | 5.0 | 13.1 | 13.3 | 10.2 | 11.0 | 10.2 | 10.0 | 12.8 |
| 2zzr | 9.9 | 10.1 | 10.7 | 9.8 | 10.6 | 12.4 | 9.4 | 6.3 | 11.1 | 10.1 | 9.8 | 11.0 | 9.6 | 15.3 | 42.3 | 100 | 18.3 | 13.6 | 16.0 | 10.5 | 11.1 | 11.8 | 11.7 | 15.7 | 12.1 | 12.7 | 9.2 | 9.7 | 12.0 | 7.7 | 12.3 | 10.3 | 8.5 | 8.9 |
| 2okx | 14.0 | 12.2 | 11.7 | 12.8 | 11.2 | 11.9 | 14.0 | 9.3 | 10.8 | 11.2 | 13.2 | 11.1 | 13.5 | 13.1 | 19.5 | 18.3 | 100 | 21.9 | 51.2 | 13.1 | 13.4 | 13.1 | 11.3 | 16.5 | 12.4 | 8.5 | 11.2 | 14.2 | 12.5 | 12.3 | 11.0 | 12.1 | 10.7 | 9.9 |
| 3w5m | 10.9 | 10.0 | 12.9 | 14.7 | 8.3 | 6.9 | 12.8 | 14.3 | 12.4 | 14.4 | 17.2 | 16.4 | 14.6 | 14.0 | 10.8 | 13.6 | 21.9 | 100 | 19.3 | 12.8 | 18.5 | 15.6 | 15.0 | 12.1 | 12.9 | 12.5 | 12.6 | 11.3 | 11.4 | 12.9 | 10.1 | 10.5 | 9.0 | 11.6 |
| ALR1 | 13.6 | 11.4 | 12.9 | 13.2 | 10.8 | 11.1 | 14.0 | 11.3 | 8.9 | 8.6 | 12.6 | 11.5 | 16.7 | 11.2 | 17.3 | 16.0 | 51.2 | 19.3 | 100 | 12.2 | 11.2 | 11.9 | 10.4 | 14.4 | 10.4 | 12.0 | 6.3 | 14.2 | 15.3 | 13.3 | 10.9 | 11.1 | 11.4 | 6.7 |
| 4ufc | 13.9 | 12.7 | 13.8 | 15.4 | 7.8 | 10.5 | 9.2 | 10.0 | 11.4 | 9.7 | 10.8 | 8.9 | 13.9 | 9.2 | 9.7 | 10.5 | 13.1 | 12.8 | 12.2 | 100 | 32.0 | 36.4 | 38.0 | 12.2 | 12.8 | 15.8 | 8.9 | 8.1 | 8.2 | 8.6 | 10.3 | 12.5 | 6.5 | 10.3 |
| 2eac | 20.7 | 18.7 | 15.8 | 18.2 | 7.7 | 9.0 | 12.5 | 10.7 | 14.2 | 12.5 | 12.7 | 12.2 | 12.8 | 10.6 | 9.2 | 11.1 | 13.4 | 18.5 | 11.2 | 32.0 | 100 | 29.3 | 29.5 | 14.6 | 13.5 | 17.6 | 10.5 | 11.2 | 13.4 | 9.4 | 10.4 | 10.7 | 7.6 | 9.7 |
| ALF1 | 14.8 | 15.1 | 14.2 | 17.0 | 4.9 | 11.1 | 8.5 | 11.7 | 8.3 | 9.2 | 11.0 | 8.1 | 10.2 | 11.5 | 15.0 | 11.8 | 13.1 | 15.6 | 11.9 | 36.4 | 29.3 | 100 | 59.7 | 14.1 | 13.3 | 14.7 | 8.7 | 10.1 | 10.3 | 7.2 | 11.3 | 12.1 | 6.9 | 11.3 |
| ALF2 | 16.7 | 16.9 | 13.0 | 16.6 | 8.4 | 7.3 | 7.7 | 11.4 | 11.9 | 10.7 | 8.3 | 10.1 | 10.5 | 10.1 | 9.9 | 11.7 | 11.3 | 15.0 | 10.4 | 38.0 | 29.5 | 59.7 | 100 | 13.4 | 12.9 | 12.0 | 10.1 | 10.5 | 11.0 | 10.6 | 12.5 | 14.1 | 7.5 | 12.6 |
| 2jf4 | 12.9 | 10.9 | 11.2 | 13.1 | 4.7 | 6.7 | 10.3 | 8.0 | 9.5 | 9.2 | 9.0 | 11.6 | 9.4 | 7.8 | 15.7 | 15.7 | 16.5 | 12.1 | 14.4 | 12.2 | 14.6 | 14.1 | 13.4 | 100 | 32.7 | 10.9 | 11.6 | 8.2 | 11.2 | 6.7 | 7.1 | 7.6 | 6.2 | 12.2 |
| TRE1 | 14.9 | 11.5 | 12.3 | 13.0 | 8.8 | 16.0 | 11.8 | 8.2 | 11.3 | 10.1 | 11.1 | 14.1 | 11.7 | 10.2 | 11.2 | 12.1 | 12.4 | 12.9 | 10.4 | 12.8 | 13.5 | 13.3 | 12.9 | 32.7 | 100 | 15.7 | 9.6 | 9.8 | 10.6 | 8.8 | 7.3 | 9.2 | 7.3 | 10.0 |
| 2d8l | 14.7 | 12.4 | 11.1 | 11.8 | 8.9 | 8.9 | 7.2 | 8.2 | 11.5 | 8.7 | 7.0 | 4.0 | 8.8 | 4.1 | 7.3 | 12.7 | 8.5 | 12.5 | 12.0 | 15.8 | 17.6 | 14.7 | 12.0 | 10.9 | 15.7 | 100 | 11.5 | 10.7 | 11.0 | 11.9 | 8.9 | 11.8 | 9.7 | 6.0 |
| 3ren | 12.0 | 11.7 | 12.1 | 10.2 | 13.7 | 10.5 | 21.0 | 18.4 | 19.8 | 18.2 | 17.2 | 17.4 | 13.3 | 14.5 | 5.0 | 9.2 | 11.2 | 12.6 | 6.3 | 8.9 | 10.5 | 8.7 | 10.1 | 11.6 | 9.6 | 11.5 | 100 | 8.5 | 11.5 | 10.5 | 9.3 | 8.7 | 2.7 | 3.7 |
| 1v7x | 13.0 | 11.1 | 10.0 | 11.8 | 13.2 | 14.6 | 8.9 | 9.8 | 4.3 | 8.3 | 8.3 | 9.2 | 11.9 | 13.0 | 13.1 | 9.7 | 14.2 | 11.3 | 14.2 | 8.1 | 11.2 | 10.1 | 10.5 | 8.2 | 9.8 | 10.7 | 8.5 | 100 | 36.1 | 36.5 | 15.9 | 15.3 | 9.5 | 10.9 |
| 2cqs | 13.8 | 10.6 | 11.7 | 13.6 | 15.9 | 15.7 | 13.7 | 11.0 | 5.5 | 7.1 | 13.5 | 9.5 | 8.1 | 12.0 | 13.3 | 12.0 | 12.5 | 11.4 | 15.3 | 8.2 | 13.4 | 10.3 | 11.0 | 11.2 | 10.6 | 11.0 | 11.5 | 36.1 | 100 | 40.8 | 17.8 | 14.1 | 11.3 | 9.6 |
| CDP1 | 9.8 | 9.9 | 11.5 | 9.7 | 16.6 | 17.2 | 9.7 | 8.1 | 7.2 | 9.8 | 9.8 | 8.9 | 7.0 | 8.6 | 10.2 | 7.7 | 12.3 | 12.9 | 13.3 | 8.6 | 9.4 | 7.2 | 10.6 | 6.7 | 8.8 | 11.9 | 10.5 | 36.5 | 40.8 | 100 | 14.7 | 13.1 | 8.5 | 9.5 |
| 1h54 | 7.7 | 10.8 | 10.1 | 8.9 | 8.3 | 11.0 | 7.6 | 9.0 | 9.5 | 8.3 | 13.2 | 11.1 | 9.6 | 11.7 | 11.0 | 12.3 | 11.0 | 10.1 | 10.9 | 10.3 | 10.4 | 11.3 | 12.5 | 7.1 | 7.3 | 8.9 | 9.3 | 15.9 | 17.8 | 14.7 | 100 | 25.8 | 15.4 | 14.5 |
| NGP1 | 8.7 | 12.1 | 8.0 | 6.8 | 11.8 | 9.9 | 9.6 | 10.1 | 8.8 | 6.6 | 7.7 | 8.0 | 7.2 | 10.0 | 10.2 | 10.3 | 12.1 | 10.5 | 11.1 | 12.5 | 10.7 | 12.1 | 14.1 | 7.6 | 9.2 | 11.8 | 8.7 | 15.3 | 14.1 | 13.1 | 25.8 | 100 | 15.6 | 13.3 |
| 1fp3 | 7.7 | 4.1 | 7.2 | 7.3 | 13.1 | 9.4 | 5.1 | 7.9 | 3.0 | 5.0 | 9.1 | 9.7 | 8.0 | 6.5 | 10.0 | 8.5 | 10.7 | 9.0 | 11.4 | 6.5 | 7.6 | 6.9 | 7.5 | 6.2 | 7.3 | 9.7 | 2.7 | 9.5 | 11.3 | 8.5 | 15.4 | 15.6 | 100 | 38.6 |
| 2gz6 | 8.4 | 7.6 | 6.7 | 7.0 | 5.6 | 6.5 | 3.1 | 8.0 | 6.1 | 5.1 | 10.6 | 9.0 | 9.5 | 8.8 | 12.8 | 8.9 | 9.9 | 11.6 | 6.7 | 10.3 | 9.7 | 11.3 | 12.6 | 12.2 | 10.0 | 6.0 | 3.7 | 10.9 | 9.6 | 9.5 | 14.5 | 13.3 | 38.6 | 100 |

The black outlined regions highlight the proteins within a subclass. A sequence identity greater than 35% is colored orange and as the percentage increases, it become red (>60%). Lower sequence identity is indicated by the colors yellow (>20%) and green (>10%).

**Table S5. Sequence identity matrix of the 19 proteins of known function in the CAL/G superfamily using the multiple sequence alignment program Clustal Omega.**

| PDB ID | 1m4w | 1h4g | 1bcx | 1uu4 | 1h8v | 2nlr | 1z3t | 1dy4 | 2rfw | 2ayh | 1dyp | 3ilf | 2vy0 | 1mve | 1uai | 1j1t | 1vav | 2ifr | 1y43 |
| --- | --- | --- | --- | --- | --- | --- | --- | --- | --- | --- | --- | --- | --- | --- | --- | --- | --- | --- | --- |
| 1m4w | 100 | 52.3 | 58.7 | 16.9 | 20.6 | 21.4 | 17.4 | 19.2 | 14.1 | 10.6 | 11.5 | 8.4 | 8.1 | 11.0 | 12.2 | 10.0 | 10.6 | 17.3 | 19.4 |
| 1h4g | 52.3 | 100 | 47.6 | 15.5 | 19.9 | 17.4 | 12.8 | 13.9 | 15.1 | 11.3 | 8.0 | 10.4 | 7.9 | 7.8 | 12.7 | 10.1 | 7.5 | 14.6 | 17.4 |
| 1bcx | 58.7 | 47.6 | 100 | 17.0 | 20.2 | 16.3 | 16.2 | 15.3 | 19.7 | 10.3 | 11.8 | 12.2 | 12.1 | 8.8 | 9.3 | 8.1 | 10.6 | 18.1 | 18.9 |
| 1uu4 | 16.9 | 15.5 | 17.0 | 100 | 43.2 | 27.0 | 9.0 | 17.1 | 9.7 | 9.9 | 10.5 | 11.8 | 11.1 | 7.4 | 8.0 | 10.4 | 17.1 | 12.9 | 14.7 |
| 1h8v | 20.6 | 19.9 | 20.2 | 43.2 | 100 | 29.8 | 10.7 | 14.9 | 11.7 | 12.4 | 13.7 | 14.8 | 11.3 | 9.7 | 12.0 | 6.4 | 12.8 | 17.5 | 15.1 |
| 2nlr | 21.4 | 17.4 | 16.3 | 27.0 | 29.8 | 100 | 14.8 | 17.2 | 11.8 | 9.7 | 8.7 | 12.6 | 9.5 | 14.1 | 15.3 | 12.0 | 12.4 | 17.7 | 20.1 |
| 1z3t | 17.4 | 12.8 | 16.2 | 9.0 | 10.7 | 14.8 | 100 | 56.4 | 49.2 | 9.8 | 13.5 | 12.2 | 12.6 | 12.2 | 17.7 | 20.7 | 20.7 | 15.4 | 13.8 |
| 1dy4 | 19.2 | 13.9 | 15.3 | 17.1 | 14.9 | 17.2 | 56.4 | 100 | 50.3 | 14.9 | 17.8 | 13.3 | 11.9 | 14.1 | 20.1 | 22.9 | 23.9 | 16.0 | 15.4 |
| 2rfw | 14.1 | 15.1 | 19.7 | 9.7 | 11.7 | 11.8 | 49.2 | 50.3 | 100 | 15.4 | 11.1 | 11.9 | 11.1 | 8.9 | 17.9 | 18.8 | 18.8 | 15.4 | 14.8 |
| 2ayh | 10.6 | 11.3 | 10.3 | 9.9 | 12.4 | 9.7 | 9.8 | 14.9 | 15.4 | 100 | 26.2 | 23.9 | 20.9 | 30.4 | 15.9 | 9.1 | 10.6 | 5.9 | 7.7 |
| 1dyp | 11.5 | 8.0 | 11.8 | 10.5 | 13.7 | 8.7 | 13.5 | 17.8 | 11.1 | 26.2 | 100 | 22.9 | 26.0 | 22.5 | 14.5 | 8.7 | 16.3 | 15.8 | 14.7 |
| 3ilf | 8.4 | 10.4 | 12.2 | 11.8 | 14.8 | 12.6 | 12.2 | 13.3 | 11.9 | 23.9 | 22.9 | 100 | 22.9 | 20.3 | 8.5 | 9.9 | 14.0 | 12.1 | 13.7 |
| 2vy0 | 8.1 | 7.9 | 12.1 | 11.1 | 11.3 | 9.5 | 12.6 | 11.9 | 11.1 | 20.9 | 26.0 | 22.9 | 100 | 16.6 | 11.1 | 10.3 | 13.7 | 10.2 | 11.1 |
| 1mve | 11.0 | 7.8 | 8.8 | 7.4 | 9.7 | 14.1 | 12.2 | 14.1 | 8.9 | 30.4 | 22.5 | 20.3 | 16.6 | 100 | 9.2 | 12.1 | 8.2 | 8.2 | 12.8 |
| 1uai | 12.2 | 12.7 | 9.3 | 8.0 | 12.0 | 15.3 | 17.7 | 20.1 | 17.9 | 15.9 | 14.5 | 8.5 | 11.1 | 9.2 | 100 | 19.5 | 27.5 | 11.1 | 10.7 |
| 1j1t | 10.0 | 10.1 | 8.1 | 10.4 | 6.4 | 12.0 | 20.7 | 22.9 | 18.8 | 9.1 | 8.7 | 9.9 | 10.3 | 12.1 | 19.5 | 100 | 18.7 | 8.0 | 8.9 |
| 1vav | 10.6 | 7.5 | 10.6 | 17.1 | 12.8 | 12.4 | 20.7 | 23.9 | 18.8 | 10.6 | 16.3 | 14.0 | 13.7 | 8.2 | 27.5 | 18.7 | 100 | 12.4 | 10.2 |
| 2ifr | 17.3 | 14.6 | 18.1 | 12.9 | 17.5 | 17.7 | 15.4 | 16.0 | 15.4 | 5.9 | 15.8 | 12.1 | 10.2 | 8.2 | 11.1 | 8.0 | 12.4 | 100 | 52.0 |
| 1y43 | 19.4 | 17.4 | 18.9 | 14.7 | 15.1 | 20.1 | 13.8 | 15.4 | 14.8 | 7.7 | 14.7 | 13.7 | 11.1 | 12.8 | 10.7 | 8.9 | 10.2 | 52.0 | 100 |

The black outlined regions highlight the proteins within a subclass. A sequence identity greater than 35% is colored orange and as the percentage increases, it become red (>60%). Lower sequence identity is indicated by the colors yellow (>20%) and green (>10%).

**Table S6. Normalized SALSA match scores for the 31 proteins of known function with the Chemical Signatures of the nine known functional families in the RPBB superfamily.**

|  | IGPS | TrpA | PRAI | HisA | HisF | RPE | OMPDC | KGPDC | HPS |
| --- | --- | --- | --- | --- | --- | --- | --- | --- | --- |
| N | 12 | 11 | 7 | 9 | 8 | 11 | 10 | 13 | 11 |
| 1pii:N | 1.00 | -0.08 | -0.20 | -0.08 | -0.09 | -0.23 | -0.20 | -0.08 | -0.14 |
| 1i4n | 1.00 | -0.15 | -0.22 | -0.18 | -0.20 | -0.23 | -0.22 | -0.08 | -0.14 |
| 2c3z | 1.00 | -0.09 | -0.22 | -0.14 | -0.13 | -0.21 | -0.22 | -0.08 | -0.14 |
| 1geq | -0.27 | 1.00 | -0.35 | -0.24 | -0.26 | -0.18 | -0.30 | -0.01 | 0.08 |
| 1qop | -0.23 | 1.00 | -0.35 | -0.22 | -0.22 | -0.14 | -0.33 | -0.03 | 0.08 |
| 1xc4 | -0.23 | 1.00 | -0.35 | -0.22 | -0.22 | -0.14 | -0.33 | -0.03 | 0.08 |
| 1rd5 | -0.29 | 0.94 | -0.33 | -0.18 | -0.20 | -0.18 | -0.32 | -0.04 | 0.06 |
| 1pii:C | -0.34 | -0.25 | 1.00 | -0.26 | 0.07 | -0.14 | 0.22 | -0.01 | -0.27 |
| 1lbm | -0.31 | -0.23 | 1.00 | -0.18 | 0.15 | -0.03 | 0.07 | -0.11 | -0.34 |
| 1qo2 | -0.24 | -0.28 | -0.07 | 0.88 | 0.59 | -0.09 | -0.02 | 0.00 | -0.09 |
| 1vzw | -0.23 | -0.15 | -0.09 | 1.00 | 0.65 | -0.05 | 0.05 | 0.18 | 0.05 |
| 2y85 | -0.23 | -0.15 | -0.09 | 1.00 | 0.65 | -0.05 | 0.05 | 0.18 | 0.05 |
| 1thf | -0.32 | -0.23 | 0.15 | 0.61 | 1.00 | -0.27 | 0.05 | -0.03 | -0.19 |
| 1h5y | -0.27 | -0.22 | 0.22 | 0.59 | 1.00 | -0.30 | 0.10 | -0.01 | -0.17 |
| 1ox6 | -0.10 | -0.28 | 0.02 | 0.43 | 0.89 | -0.30 | -0.10 | -0.10 | -0.13 |
| 1rpx | -0.27 | -0.02 | 0.02 | -0.14 | -0.35 | 1.00 | 0.13 | 0.07 | -0.17 |
| 2fli | -0.27 | -0.02 | 0.02 | -0.08 | -0.28 | 1.00 | 0.07 | 0.01 | -0.17 |
| 1h1y | -0.24 | -0.02 | 0.04 | -0.12 | -0.33 | 1.00 | 0.03 | 0.01 | -0.13 |
| 1tqj | -0.27 | -0.02 | 0.02 | -0.10 | -0.30 | 1.00 | 0.07 | 0.01 | -0.17 |
| 3ovp | -0.24 | -0.02 | 0.02 | -0.08 | -0.28 | 1.00 | 0.07 | 0.03 | -0.16 |
| 1dbt | -0.31 | -0.09 | 0.15 | -0.14 | -0.07 | 0.06 | 1.00 | 0.62 | 0.36 |
| 1dv7 | -0.36 | -0.12 | 0.13 | -0.14 | -0.02 | -0.08 | 0.90 | 0.60 | 0.33 |
| 1dqw | -0.31 | -0.23 | 0.13 | -0.14 | -0.04 | 0.05 | 1.00 | 0.45 | 0.19 |
| 1l2u | -0.31 | -0.05 | 0.15 | -0.16 | -0.09 | -0.02 | 1.00 | 0.62 | 0.36 |
| 2za1 | -0.34 | -0.26 | -0.09 | -0.22 | -0.15 | -0.18 | 0.78 | 0.44 | 0.14 |
| 3qw3 | -0.32 | -0.28 | -0.13 | -0.18 | -0.11 | -0.20 | 0.73 | 0.38 | 0.11 |
| 3l0k | -0.31 | -0.23 | 0.11 | -0.16 | -0.04 | 0.00 | 1.00 | 0.45 | 0.20 |
| 1xbv | 0.00 | 0.06 | -0.07 | -0.02 | 0.04 | -0.24 | 0.52 | 1.00 | 0.78 |
| 3exr | 0.02 | 0.08 | -0.04 | -0.04 | 0.00 | -0.23 | 0.50 | 1.00 | 0.81 |
| 3ajx | -0.19 | 0.14 | -0.09 | -0.16 | -0.04 | -0.24 | 0.50 | 0.80 | 1.00 |
| HPS1 | -0.21 | 0.14 | -0.09 | -0.14 | -0.02 | -0.26 | 0.40 | 0.80 | 1.00 |

N is number of aligned spatial positions in the Chemical Signature of each functional family. Table shows function promiscuity across different functional classes (green on the off-diagonal).

**Table S7. Normalized SALSA match scores for the 44 SG proteins against the Chemical Signatures of the nine known functional families in the RPBB superfamily.**

|  | IGPS | TrpA | PRAI | HisA | HisF | RPE | OMPDC | KGPDC | HPS |
| --- | --- | --- | --- | --- | --- | --- | --- | --- | --- |
| N | 12 | 11 | 7 | 9 | 8 | 11 | 10 | 13 | 11 |
| Range -Knowns | 1.00 – 1.00 | 0.94 - 1.00 | X | 0.88 - 1.00 | 0.89 – 1.00 | 1.00 – 1.00 | 0.73 - 1.00 | X | X |
| 1vc4 | 1.00 | -0.15 | -0.07 | -0.18 | -0.17 | -0.24 | -0.22 | -0.08 | -0.14 |
| 3qja | 1.00 | -0.15 | -0.07 | -0.18 | -0.17 | -0.23 | -0.20 | -0.06 | -0.13 |
| 3tsm | 1.00 | -0.17 | -0.20 | -0.10 | -0.11 | -0.26 | -0.20 | -0.07 | -0.14 |
| 1j5t | 0.95 | -0.15 | -0.20 | -0.18 | -0.17 | -0.23 | -0.22 | -0.10 | -0.16 |
| 1ujp | -0.29 | 0.82 | -0.33 | -0.20 | -0.20 | -0.17 | -0.10 | 0.04 | 0.05 |
| 2e09 | -0.27 | 0.71 | -0.39 | -0.24 | -0.26 | -0.18 | -0.30 | -0.01 | 0.03 |
| 1wq5 | -0.36 | 0.75 | -0.41 | -0.24 | -0.24 | -0.14 | -0.33 | -0.03 | 0.08 |
| 3tha | -0.34 | 0.68 | -0.37 | -0.28 | -0.28 | -0.09 | -0.32 | -0.04 | 0.06 |
| 3nav | -0.23 | 1.00 | -0.35 | -0.20 | -0.20 | -0.14 | -0.33 | -0.03 | 0.08 |
| 5kzm | -0.24 | 1.00 | -0.35 | -0.20 | -0.20 | -0.14 | -0.33 | -0.03 | 0.08 |
| 5kmy | -0.27 | 0.89 | -0.35 | -0.18 | -0.15 | -0.18 | -0.20 | 0.06 | 0.08 |
| 5kin | -0.29 | 0.95 | -0.35 | -0.24 | -0.26 | -0.17 | -0.32 | -0.04 | 0.05 |
| 5k9x | -0.27 | 0.82 | -0.35 | -0.18 | -0.15 | -0.18 | -0.20 | 0.06 | 0.06 |
| 1v5x | -0.34 | -0.22 | 1.00 | -0.26 | 0.07 | -0.09 | 0.08 | -0.11 | -0.25 |
| 4wui | -0.31 | -0.22 | 1.00 | -0.22 | 0.11 | -0.09 | 0.03 | -0.15 | -0.30 |
| 4gj1 | -0.26 | -0.15 | -0.09 | 0.90 | 0.65 | -0.06 | 0.03 | 0.18 | 0.02 |
| 4x2r | -0.23 | -0.29 | -0.09 | 1.00 | 0.65 | -0.05 | -0.02 | 0.00 | -0.09 |
| 4x9s | -0.23 | -0.29 | -0.09 | 1.00 | 0.65 | -0.05 | -0.02 | 0.00 | -0.09 |
| 4wd0 | -0.23 | -0.29 | -0.09 | 1.00 | 0.65 | -0.05 | -0.02 | 0.00 | -0.09 |
| 4u28 | -0.23 | -0.29 | -0.09 | 1.00 | 0.65 | -0.05 | -0.02 | 0.00 | -0.09 |
| 2a0n | -0.31 | -0.20 | 0.15 | 0.45 | 0.65 | -0.27 | 0.05 | 0.06 | -0.11 |
| 1ka9 | -0.32 | -0.20 | 0.15 | 0.61 | 1.00 | -0.26 | 0.05 | -0.03 | -0.19 |
| 4nu7 | -0.26 | -0.06 | 0.02 | -0.08 | -0.28 | 1.00 | 0.07 | 0.03 | -0.16 |
| 1tqx | -0.24 | -0.06 | 0.04 | -0.10 | -0.30 | 1.00 | -0.02 | -0.03 | -0.13 |
| 3cu2 | -0.31 | 0.02 | -0.20 | -0.14 | -0.39 | 0.68 | -0.15 | -0.06 | -0.13 |
| 3inp | -0.26 | -0.02 | 0.04 | -0.06 | -0.26 | 0.94 | -0.02 | -0.04 | -0.14 |
| 3qc3 | -0.24 | -0.02 | 0.02 | -0.10 | -0.30 | 0.94 | -0.02 | -0.04 | -0.14 |
| 2aqw | -0.10 | -0.19 | -0.02 | -0.12 | -0.13 | -0.14 | 0.70 | 0.41 | 0.23 |
| 3ldv | -0.31 | -0.05 | 0.24 | -0.16 | -0.09 | -0.02 | 1.00 | 0.62 | 0.36 |
| 1vqt | -0.34 | -0.05 | 0.20 | -0.16 | -0.07 | -0.06 | 0.90 | 0.60 | 0.33 |
| 2ffc | -0.39 | -0.17 | -0.11 | -0.20 | -0.15 | -0.12 | 0.78 | 0.49 | 0.20 |
| 2cze | -0.32 | -0.15 | 0.17 | -0.04 | -0.04 | -0.03 | 0.90 | 0.53 | 0.42 |
| 4mjz | -0.32 | -0.28 | -0.09 | -0.20 | -0.11 | -0.18 | 0.78 | 0.38 | 0.11 |
| 3tfx | -0.32 | -0.12 | 0.15 | -0.10 | -0.02 | 0.03 | 1.00 | 0.62 | 0.36 |
| 3ru6 | -0.31 | -0.08 | 0.15 | -0.20 | -0.13 | 0.03 | 1.00 | 0.62 | 0.36 |
| 2f84 | -0.34 | -0.23 | -0.09 | -0.22 | -0.15 | -0.15 | 0.78 | 0.47 | 0.17 |
| 2yyu | -0.31 | -0.09 | 0.15 | -0.14 | -0.07 | 0.06 | 1.00 | 0.62 | 0.36 |
| 3r89 | -0.36 | -0.28 | -0.13 | -0.18 | -0.13 | -0.11 | 0.78 | 0.36 | 0.08 |
| 3v75 | -0.27 | -0.28 | -0.13 | -0.14 | -0.07 | -0.20 | 0.73 | 0.36 | 0.08 |
| 3ieb | -0.02 | 0.08 | 0.02 | 0.00 | 0.04 | -0.15 | 0.53 | 1.00 | 0.78 |
| 3f4w | -0.16 | 0.15 | -0.07 | -0.08 | 0.02 | -0.26 | 0.27 | 0.69 | 1.00 |
| 2agk | -0.24 | -0.12 | 0.11 | 0.12 | 0.20 | -0.11 | -0.05 | 0.07 | 0.06 |
| 1y0e | 0.08 | -0.22 | -0.11 | -0.10 | -0.15 | -0.03 | -0.08 | 0.01 | 0.09 |
| 1yxy | 0.08 | -0.22 | -0.11 | -0.04 | -0.09 | -0.03 | -0.08 | 0.01 | 0.03 |

N is number of aligned spatial positions in the Chemical Signature of each functional family. The next row shows the range of scores between the previously characterized members of the family, for families with more than two previously characterized members. Cells with negative scores are colored shades of red; cells with positive scores are colored shades of green.

**Table S8. Normalized SALSA match scores for the 34 proteins of known function with the Chemical Signatures of the nine known functional families in the 6-HG superfamily.**

|  | AGG | AMAN | EXC | CELL | UGH | ALR | ALF/ ALG | TRE | URH | AMY | CDP | NGP | NAE |
| --- | --- | --- | --- | --- | --- | --- | --- | --- | --- | --- | --- | --- | --- |
| N | 12 | 15 | 8 | 12 | 12 | 12 | 11 | 9 | 14 | 16 | 16 | 10 | 12 |
| 1gai | 1.00 | 0.51 | 0.07 | -0.23 | -0.18 | 0.20 | -0.25 | 0.66 | -0.20 | -0.17 | 0.02 | -0.01 | -0.05 |
| 1ayx | 1.00 | 0.45 | 0.00 | -0.22 | -0.15 | 0.16 | -0.16 | 0.66 | -0.15 | -0.16 | -0.03 | -0.01 | -0.06 |
| 1lf9 | 1.00 | 0.52 | 0.17 | -0.21 | -0.13 | 0.11 | -0.28 | 0.66 | -0.20 | -0.13 | 0.01 | -0.03 | -0.02 |
| 1ug9 | 1.00 | 0.45 | 0.15 | -0.18 | -0.08 | 0.18 | -0.27 | 0.66 | -0.18 | -0.22 | 0.05 | -0.06 | 0.02 |
| 3qt9 | 0.64 | 1.00 | 0.07 | -0.23 | -0.12 | 0.24 | -0.29 | 0.57 | -0.12 | -0.10 | 0.13 | -0.06 | -0.13 |
| 3qsp | 0.64 | 1.00 | 0.07 | -0.21 | -0.15 | 0.24 | -0.27 | 0.57 | -0.11 | -0.10 | 0.13 | -0.06 | -0.14 |
| 1cem | -0.23 | -0.27 | 1.00 | -0.20 | -0.16 | -0.29 | -0.32 | -0.32 | -0.12 | 0.21 | -0.18 | -0.23 | -0.30 |
| 1wu4 | -0.25 | -0.26 | 1.00 | -0.12 | -0.15 | -0.18 | -0.28 | -0.34 | -0.09 | 0.13 | -0.25 | -0.20 | -0.27 |
| 1v5c | -0.17 | -0.21 | 0.59 | -0.20 | -0.05 | -0.28 | -0.12 | -0.36 | -0.14 | 0.06 | -0.23 | -0.21 | -0.20 |
| 1h12 | -0.24 | -0.25 | 0.89 | -0.16 | -0.17 | -0.24 | -0.28 | -0.34 | -0.15 | 0.15 | -0.26 | -0.29 | -0.29 |
| 1kfg | -0.20 | -0.37 | 0.04 | 1.00 | -0.01 | -0.03 | -0.19 | -0.24 | 0.01 | -0.20 | -0.37 | -0.03 | -0.26 |
| 1clc | -0.09 | -0.28 | -0.22 | 0.76 | -0.01 | 0.13 | -0.02 | 0.03 | -0.06 | -0.20 | -0.39 | 0.06 | -0.35 |
| 1ksc | -0.19 | -0.33 | 0.17 | 0.95 | 0.01 | -0.02 | -0.27 | -0.21 | 0.06 | -0.16 | -0.37 | 0.01 | -0.22 |
| 1ia6 | -0.18 | -0.32 | 0.07 | 1.00 | 0.00 | -0.02 | -0.21 | -0.23 | -0.03 | -0.20 | -0.34 | 0.00 | -0.35 |
| 2d5j | -0.11 | -0.26 | 0.00 | 0.01 | 1.00 | -0.19 | -0.18 | -0.11 | 0.19 | -0.13 | -0.18 | 0.03 | -0.14 |
| 2zzr | -0.11 | -0.30 | 0.00 | 0.10 | 1.00 | -0.09 | -0.03 | -0.11 | 0.23 | -0.12 | -0.21 | 0.07 | -0.09 |
| 2okx | 0.12 | -0.03 | -0.26 | 0.01 | -0.25 | 1.00 | -0.06 | 0.13 | -0.27 | -0.21 | -0.20 | 0.23 | -0.13 |
| 3w5m | -0.05 | -0.09 | -0.28 | -0.07 | -0.29 | 0.72 | -0.12 | -0.08 | -0.35 | -0.21 | -0.24 | 0.29 | -0.24 |
| ALR1 | 0.12 | -0.03 | -0.26 | 0.01 | -0.25 | 1.00 | -0.06 | 0.13 | -0.27 | -0.21 | -0.20 | 0.23 | -0.13 |
| 4ufc | -0.16 | -0.26 | -0.30 | -0.17 | -0.17 | -0.19 | 0.81 | -0.29 | -0.15 | -0.22 | -0.20 | 0.04 | 0.08 |
| 2eac | -0.16 | -0.25 | -0.26 | -0.26 | -0.21 | -0.19 | 0.94 | -0.18 | -0.03 | -0.21 | -0.22 | 0.04 | 0.04 |
| ALF1 | -0.03 | -0.14 | -0.37 | -0.15 | -0.22 | -0.18 | 1.00 | -0.23 | -0.09 | -0.24 | -0.21 | 0.09 | 0.04 |
| ALF2 | -0.21 | -0.29 | -0.33 | -0.15 | -0.22 | -0.17 | 1.00 | -0.21 | -0.08 | -0.21 | -0.25 | 0.09 | -0.14 |
| 2jf4 | 0.43 | 0.26 | -0.09 | -0.02 | 0.09 | 0.18 | -0.24 | 1.00 | -0.15 | -0.26 | -0.03 | 0.21 | 0.05 |
| TRE1 | 0.43 | 0.29 | -0.13 | 0.09 | 0.21 | 0.10 | -0.28 | 1.00 | -0.16 | -0.23 | -0.03 | 0.14 | -0.08 |
| 2d8l | -0.20 | -0.17 | -0.07 | 0.09 | 0.41 | -0.30 | 0.06 | -0.11 | 1.00 | -0.16 | -0.27 | -0.16 | -0.24 |
| 3ren | -0.16 | -0.11 | 0.41 | -0.15 | -0.13 | -0.17 | -0.19 | -0.16 | -0.16 | 1.00 | -0.19 | 0.00 | -0.30 |
| 1v7x | 0.08 | 0.17 | 0.04 | -0.32 | -0.13 | -0.10 | -0.19 | 0.02 | -0.25 | -0.24 | 1.00 | -0.21 | -0.17 |
| 2cqs | 0.08 | 0.22 | -0.02 | -0.31 | -0.11 | -0.09 | -0.19 | 0.02 | -0.25 | -0.27 | 1.00 | -0.21 | -0.20 |
| CDP1 | 0.07 | 0.32 | 0.02 | -0.31 | -0.05 | -0.08 | -0.21 | 0.05 | -0.21 | -0.26 | 0.87 | -0.23 | -0.16 |
| 1h54 | 0.01 | -0.23 | -0.28 | 0.18 | 0.20 | 0.12 | 0.06 | -0.07 | -0.03 | -0.23 | -0.27 | 1.00 | -0.14 |
| NGP1 | -0.05 | -0.24 | -0.30 | -0.12 | -0.11 | 0.03 | -0.09 | -0.19 | -0.11 | -0.15 | -0.25 | 0.94 | -0.16 |
| 1fp3 | -0.28 | -0.34 | -0.33 | -0.31 | -0.03 | -0.22 | -0.21 | -0.52 | -0.33 | -0.49 | -0.36 | -0.23 | 1.00 |
| 2gz6 | -0.28 | -0.34 | -0.33 | -0.31 | -0.03 | -0.22 | -0.21 | -0.52 | -0.33 | -0.49 | -0.36 | -0.23 | 1.00 |

N is the number of aligned spatial positions in the Chemical Signature of each functional family. Table shows function promiscuity across different functional classes (green on the off-diagonal).

**Table S9. Normalized SALSA match scores for the 11 SG proteins against the Chemical Signatures of the 13 known functional families in the 6-HG superfamily.**

|  | AGG | AMAN | EXC | CELL | UGH | ALR | ALF/ ALG | TRE | URH | AMY | CDP | NGP | NAE |
| --- | --- | --- | --- | --- | --- | --- | --- | --- | --- | --- | --- | --- | --- |
| N | 12 | 15 | 8 | 12 | 12 | 12 | 11 | 9 | 14 | 16 | 16 | 10 | 12 |
| Range - Knowns | 1.00 - 1.00 | X | 0.59 - 1.00 | 0.76 - 1.00 | X | 0.72 - 1.00 | 0.81 - 1.00 | X | X | X | 0.87 - 1.00 | X | X |
| 5goo | 0.54 | 0.24 | -0.02 | -0.16 | -0.14 | 0.04 | -0.19 | 0.32 | -0.18 | -0.11 | 0.04 | -0.03 | -0.17 |
| 2p0v | 0.64 | 0.96 | 0.07 | -0.18 | -0.18 | 0.24 | -0.27 | 0.57 | -0.06 | -0.12 | 0.13 | -0.06 | -0.11 |
| 3on6 | 0.64 | 0.96 | 0.07 | -0.18 | -0.18 | 0.24 | -0.27 | 0.57 | -0.06 | -0.12 | 0.13 | -0.06 | -0.11 |
| 3pmm | -0.25 | -0.28 | -0.09 | -0.01 | 0.51 | -0.28 | -0.09 | -0.11 | 0.37 | -0.13 | -0.31 | -0.07 | -0.13 |
| 3qwt | -0.19 | -0.19 | -0.09 | 0.01 | 0.51 | -0.28 | -0.09 | -0.11 | 0.37 | -0.13 | -0.31 | -0.07 | -0.13 |
| 3cih | -0.08 | -0.11 | -0.26 | 0.16 | -0.21 | 0.83 | 0.10 | -0.05 | -0.21 | -0.18 | -0.07 | 0.19 | -0.09 |
| 2rdy | -0.17 | -0.27 | -0.33 | -0.21 | -0.17 | -0.20 | 0.81 | -0.27 | -0.19 | -0.27 | -0.23 | -0.01 | 0.07 |
| 2afa | -0.35 | -0.39 | -0.39 | -0.31 | -0.07 | -0.08 | -0.16 | -0.40 | -0.25 | -0.50 | -0.34 | -0.13 | 0.56 |
| 3gt5 | -0.35 | -0.38 | -0.33 | -0.27 | -0.20 | -0.17 | -0.18 | -0.45 | -0.26 | -0.43 | -0.21 | -0.30 | 0.56 |
| 3k7x | -0.28 | -0.44 | -0.09 | -0.37 | 0.12 | -0.43 | -0.18 | -0.45 | -0.03 | -0.27 | -0.37 | -0.17 | 0.04 |
| 4mu9 | -0.20 | -0.25 | -0.20 | -0.28 | 0.07 | -0.31 | -0.19 | -0.19 | -0.18 | -0.07 | -0.33 | -0.09 | 0.19 |

N is number of aligned spatial positions in the Chemical Signature of each functional family. The next row shows the range of scores between the previously characterized members of the family, for families with more than two previously characterized members. Cells with negative scores are colored shades of red; cells with positive scores are colored shades of green.

**Table S10. Normalized SALSA match scores for the 19 proteins of known function with the Chemical Signatures of the nine known functional families in the CAL/G superfamily.**

|  | XYL | ENDO | CBH | GH16 | ALY | PEP |
| --- | --- | --- | --- | --- | --- | --- |
| N | 14 | 10 | 16 | 9 | 10 | 11 |
| 1m4w | 1.00 | -0.03 | -0.39 | -0.15 | -0.14 | -0.46 |
| 1h4g | 0.97 | 0.00 | -0.38 | -0.19 | -0.18 | -0.34 |
| 1bcx | 1.00 | -0.02 | -0.41 | -0.15 | -0.16 | -0.46 |
| 1uu4 | -0.14 | 1.00 | -0.28 | -0.27 | -0.12 | -0.41 |
| 1h8v | -0.14 | 1.00 | -0.31 | -0.27 | -0.12 | -0.41 |
| 2nlr | -0.03 | 0.80 | -0.33 | -0.27 | -0.23 | -0.29 |
| 1z3t | -0.20 | -0.07 | 1.00 | 0.08 | -0.23 | -0.34 |
| 1dy4 | -0.19 | -0.07 | 1.00 | 0.13 | -0.21 | -0.32 |
| 2rfw | -0.18 | -0.07 | 0.92 | 0.13 | -0.23 | -0.32 |
| 2ayh | -0.28 | -0.34 | -0.20 | 0.64 | -0.25 | -0.37 |
| 1dyp | -0.17 | -0.23 | -0.13 | 0.66 | -0.28 | -0.27 |
| 3ilf | -0.28 | -0.25 | -0.17 | 0.72 | -0.19 | -0.40 |
| 2vy0 | -0.35 | -0.33 | -0.14 | 0.60 | -0.33 | -0.43 |
| 1mve | -0.24 | -0.39 | -0.28 | 0.57 | -0.32 | -0.34 |
| 1uai | -0.35 | -0.44 | -0.38 | -0.12 | 0.88 | -0.34 |
| 1j1t | -0.35 | -0.33 | -0.43 | -0.16 | 1.00 | -0.40 |
| 1vav | -0.34 | -0.41 | -0.45 | -0.25 | 1.00 | -0.31 |
| 2ifr | -0.47 | -0.49 | -0.50 | -0.10 | -0.51 | 1.00 |
| 1y43 | -0.55 | -0.62 | -0.51 | -0.15 | -0.54 | 0.94 |

N is the number of aligned spatial positions in the Chemical Signature of each functional family. Table shows no function promiscuity across different functional classes (no green on the off-diagonal).

**Table S11. Normalized SALSA match scores for the eight SG proteins against the Chemical Signatures of the six known functional families in the CAL/G superfamily.**

|  | XYL | ENDO | CBH | GH16 | ALY | PEP |
| --- | --- | --- | --- | --- | --- | --- |
| N | 14 | 10 | 16 | 9 | 10 | 11 |
| Range - Knowns | 0.97 - 1.00 | 0.80 - 1.00 | 0.92 - 1.00 | 0.60 - 0.72 | 0.88 - 1.00 | X |
| 3rq0 | -0.23 | -0.20 | -0.19 | 0.40 | -0.26 | -0.35 |
| 1y7b | -0.08 | -0.15 | -0.31 | -0.19 | -0.32 | -0.41 |
| 1yif | -0.27 | -0.30 | -0.28 | -0.15 | -0.32 | -0.31 |
| 1yrz | -0.08 | -0.26 | -0.40 | -0.09 | -0.35 | -0.52 |
| 3h3l | -0.24 | -0.34 | -0.41 | -0.30 | -0.09 | -0.29 |
| 3hbk | -0.36 | -0.33 | -0.36 | -0.10 | -0.40 | -0.28 |
| 3nmb | -0.35 | -0.36 | -0.30 | 0.09 | -0.30 | -0.09 |
| 3osd | -0.36 | -0.34 | -0.37 | -0.18 | -0.33 | -0.31 |

N is number of aligned spatial positions in the Chemical Signature of each functional family. The next row shows the range of scores between the previously characterized members of the family, for families with more than two previously characterized members. Cells with negative scores are colored shades of red; cells with positive scores are colored shades of green.

**Table S12. Structural Genomics protein source list.**

| **PDB ID** | **Label** | **Species** | **Resolution (**Å) | **Center** | **Annotation** | | |
| --- | --- | --- | --- | --- | --- | --- | --- |
|  |  |  |  |  | **Original** | **SALSA** | **GRASP-Func** |
| ***Ribulose Phosphate Binding Barrel (RPBB) Superfamily*** | | | | | | | |
| **1vc4** | R1 | *Thermus thermophilus* | 1.80 | RIKEN | Putative IGPS | IGPS (s) | IGPS |
| **3qja** | R2 | *Mycobacterium tuberculosis* | 1.29 | TBSGC | Putative IGPS | IGPS (s) | IGPS |
| **3tsm** | R3 | *Brucella melitensis* | 2.15 | SSGCID | Putative IGPS | IGPS (s) | IGPS |
| **1j5t** | R4 | *Thermotoga maritima* | 3.00 | JCSG | Putative IGPS | IGPS (s) | IGPS |
| **1ujp** | R5 | *Thermus thermophilus* | 1.34 | RIKEN | Putative TrpA | TrpA (m) | TrpA |
| **2e09** | R6 | *Pyrococcus furiosus* | 2.40 | RIKEN | Putative TrpA | TrpA (m) | TrpA |
| **1wq5** | R7 | *Escherichia coli* | 2.30 | RIKEN | Putative TrpA | TrpA (m) | TrpA |
| **3tha** | R8 | *Campylobacter jejuni* | 2.37 | CSGID | Putative TrpA | TrpA (w) | TrpA |
| **3nav** | R9 | *Vibrio cholerae* | 2.10 | CSGID | Putative TrpA | TrpA (s) | TrpA |
| **5kzm** | R10 | *Francisella tularensis* | 2.80 | CSGID | Putative TrpA | TrpA (s) | TrpA |
| **5kmy** | R11 | *Legionella pneumophila* | 1.91 | CSGID | Putative TrpA | TrpA (m) | TrpA |
| **5kin** | R12 | *Streptococcus pneumoniae* | 2.45 | CSGID | Putative TrpA | TrpA (s) | TrpA |
| **5k9x** | R13 | *Legionella pneumophila* | 2.02 | CSGID | Putative TrpA | TrpA (m) | TrpA |
| **1v5x** | R14 | *Thermus thermophilus* | 2.00 | RIKEN | Putative PRAI | PRAI (s) | PRAI |
| **4wui** | R15 | *Jonesia denitrificans* | 1.09 | MCSG | Putative PRAI | PRAI (s) | PRAI |
| **4gj1** | R16 | *Campylobacter jejuni* | 2.15 | CSGID | Putative HisA | HisA (s) | HisA |
| **4x2r** | R17 | *Actinomyces urogenitalis* | 1.05 | MCSG | Putative HisA | HisA (s) | HisA |
| **4x9s** | R18 | *Streptomyces sp. Mg1* | 1.60 | MCSG | Putative HisA | HisA (s) | HisA |
| **4wd0** | R19 | *Arthrobacter aurescens* | 1.50 | MCSG | Putative HisA | HisA (s) | HisA |
| **4u28** | R20 | *Streptomyces sviceus ATCC* | 1.33 | MCSG | Putative HisA | HisA (s) | HisA |
| **2a0n** | R21 | *Thermotoga maritima* | 1.64 | JCSG | Putative HisF | HisF (w) | HisF |
| **1ka9** | R22 | *Thermus thermophilus* | 2.30 | RIKEN | Putative HisF | HisF (s) | HisF |
| **4nu7** | R23 | *Toxoplasma gondii* | 2.05 | CSGID | Putative RPE | RPE (s) | RPE |
| **1tqx** | R24 | *Plasmodium falciparum* | 2.00 | SGPPC | Putative RPE | RPE (s) | RPE |
| **3cu2** | R25 | *Haemophilus somnus* | 1.91 | JCSG | Putative RPE | RPE (w) | RPE |
| **3inp** | R26 | *Francisella tularensis* | 2.05 | CSGID | Putative RPE | RPE (s) | RPE |
| **3qc3** | R27 | *Homo sapiens* | 2.20 | JCSG | Putative RPE | RPE (s) | RPE |
| **2aqw** | R28 | *Plasmodium yoelii* | 2.00 | SGC | Putative OMPDC | OMPDC (m) | OMPDC |
| **3ldv** | R29 | *Vibrio cholerae* | 1.77 | CSGID | Putative OMPDC | OMPDC (s) | OMPDC |
| **1vqt** | R30 | *Thermotoga maritima* | 2.00 | JCSG | Putative OMPDC | OMPDC (s) | OMPDC |
| **2ffc** | R31 | *Plasmodium vivax* | 1.70 | SGC | Putative OMPDC | OMPDC (s) | OMPDC |
| **2cze** | R32 | *Pyrococcus horikoshii* | 2.00 | RIKEN | Putative OMPDC | OMPDC (s) | OMPDC |
| **4mjz** | R33 | *Toxoplasma gondii* | 2.75 | CSGID | Putative OMPDC | OMPDC (s) | OMPDC |
| **3tfx** | R34 | *Lactobacillus acidophilus* | 2.19 | NYSGRC | Putative OMPDC | OMPDC (s) | OMPDC |
| **3ru6** | R35 | *Campylobacter jejuni* | 1.80 | CSGID | Putative OMPDC | OMPDC (s) | OMPDC |
| **2f84** | R36 | *Plasmodium falciparum* | 2.10 | SGPPC | Putative OMPDC | OMPDC (s) | OMPDC |
| **2yyu** | R37 | *Geobacillus kaustophilus* | 2.20 | RIKEN | Putative OMPDC | OMPDC (s) | OMPDC |
| **3r89** | R38 | *Anaerococcus prevotii* | 1.84 | MCSG | Putative OMPDC | OMPDC (s) | OMPDC |
| **3v75** | R39 | *Streptomyces avermitilis* | 1.40 | MCSG | Putative OMPDC | OMPDC (s) | OMPDC |
| **3ieb** | R40 | *Vibrio cholerae* | 2.10 | CSGID | Putative KGPDC | KGPDC (s) | KGPDC |
| **3f4w** | R41 | *Salmonella typhimurium* | 1.65 | - | Putative HPS | HPS (s) | HPS |
| **2agk** | R42 | *Saccharomyces cerevisiae* | 1.30 | PSYSG | Putative HisA/HisF | No match | No match |
| **1y0e** | R43 | *Staphylococcus aureus* | 1.95 | MCSG | Putative ManNAc-6-P epimerase | No match | No match |
| **1yxy** | R44 | *Streptococcus pyogenes* | 1.60 | MCSG | Putative ManNAc-6-P epimerase | No match | No match |
| ***6-Hairpin Glycosidase (6-HG) Superfamily*** | | | | | | | |
| **5goo** | H1 | *Nostoc sp. PCC 7120* | 2.11 | - | Putative alkaline invertase | AGG (w) | AGG |
| **2p0v** | H2 | *Bacteroides thetaiotaomicron* | 2.10 | NSGC | Putative AMAN | AMAN (s) | AMAN |
| **3on6** | H3 | *Bacteroides ovatus* | 1.70 | JCSG | Putative AMAN | AMAN (s) | AMAN |
| **3pmm** | H4 | *Klebsiella pneumoniae* | 1.90 | MCSG | Putative GH105 Family Protein | UGH (w) | URH |
| **3qwt** | H5 | *Salmonella paratyphi* | 2.18 | MCSG | Putative GH105 Family Protein | UGH (w) | URH |
| **3cih** | H6 | *Bacteroides thetaiotaomicron* | 2.33 | NYSGXRC | Putative ALR | ALR (s) | ALR |
| **2rdy** | H7 | *Bacillus halodurans* | 2.03 | NYSGXRC | Putative ALF | ALF/ALG (s) | ALG |
| **2afa** | H8 | *Salmonella typhimurium* | 2.15 | NYSGXRC | Putative NAE | NAE (w) | NAE |
| **3gt5** | H9 | *Xylella fastidiosa* | 1.12 | CESG | Putative NAE | NAE (w) | NAE |
| **3k7x** | H10 | *Listeria innocua* serovar 6a | 1.89 | NESG | Putative GH76 Family Protein | No Match | No Match |
| **4mu9** | H11 | *Bacteroides thetaiotaomicron* | 1.89 | JCSG | Putative Glycosyl Hydrolase | No Match | No Match |
| ***Concanavalin A-like Lectins/Glucanases (CAL/G) Superfamily*** | | | | | | | |
| **3rq0** | C1 | *Mycobacterium smegmatis* | 2.02 | MCSG | Putative GH16 Family Protein | GH16 (w) | GH16 |
| **1y7b** | C2 | *Clostridium acetobutylicum* | 1.60 | NYSGXRC | Putative beta-Xylosidase | No Match | No Match |
| **1yif** | C3 | *Bacillus subtilis* | 1.80 | NYSGXRC | Putative beta-Xylosidase | No Match | No Match |
| **1yrz** | C4 | *Bacillus halodurans* | 2.00 | NYSGXRC | Putative beta-Xylosidase | No Match | No Match |
| **3h3l** | C5 | *Parabacteroides distasonis* | 1.59 | JCSG | Putative Sugar Hydrolase | No Match | No Match |
| **3hbk** | C6 | *Parabacteroides distasonis* | 2.36 | JCSG | Putative Glycosyl Hydrolase | No Match | No Match |
| **3nmb** | C7 | *Bacteroides ovatus* | 2.40 | JCSG | Putative Sugar Hydrolase | No Match | No Match |
| **3osd** | C8 | *Bacteroides thetaiotaomicron* | 1.80 | JCSG | Putative Glycosyl Hydrolase | No Match | No Match |

Consortia and Centers reporting these structures are: CESG – Center for Eukaryotic Structural Genomics; CSGID – Center for Structural Genomics of Infectious Diseases; JCSG – Joint Center for Structural Genomics; MCSG – Midwest Center for Structural Genomics; NYSGXRC – New York SGX Research Center for Structural Genomics; NYSGRC – New York Structural Genomics Research Consortium; NESG – Northeast Structural Genomics Consortium; PSYSG – Paris-Sud Yeast Structural Genomics; RIKEN – RIKEN Structural Genomics / Proteomics Initiative; SSGCID – Seattle Structural Genomics Center for Infectious Diseases; SGC – Structural Genomics Consortium; SGPPC – Structural Genomics of Pathogenic Protozoa Consortium; TBSGC – TB Structural Genomics Consortium. The *Salmonella typhimurium* structure 3f4w was reported by J. Vijayalakshmi, T.C. Meredith and R.W. Woodard (unpublished) and the *Nostoc sp. PCC 7120* structure 5goo was reported by Xie, J. et al. (ADD REF). For the cases where SALSA predicts two functions with different degrees of matching (strong vs. moderate vs. weak), the best prediction is listed.

**Fig S1. Aligned Chemical Signature residues of IGPS with bound indole-3-glycerol phosphate (IGP) in green.** Aligned structures from *E. coli* (PDB 1pii:B) in gray, *T. maritima* (PDB 1i4n) in blue, and *S. solfataricus* (PDB 2c3z) in yellow; residue numbers correspond to the *E. coli* structure. Image rendered in UCSF Chimera [37].


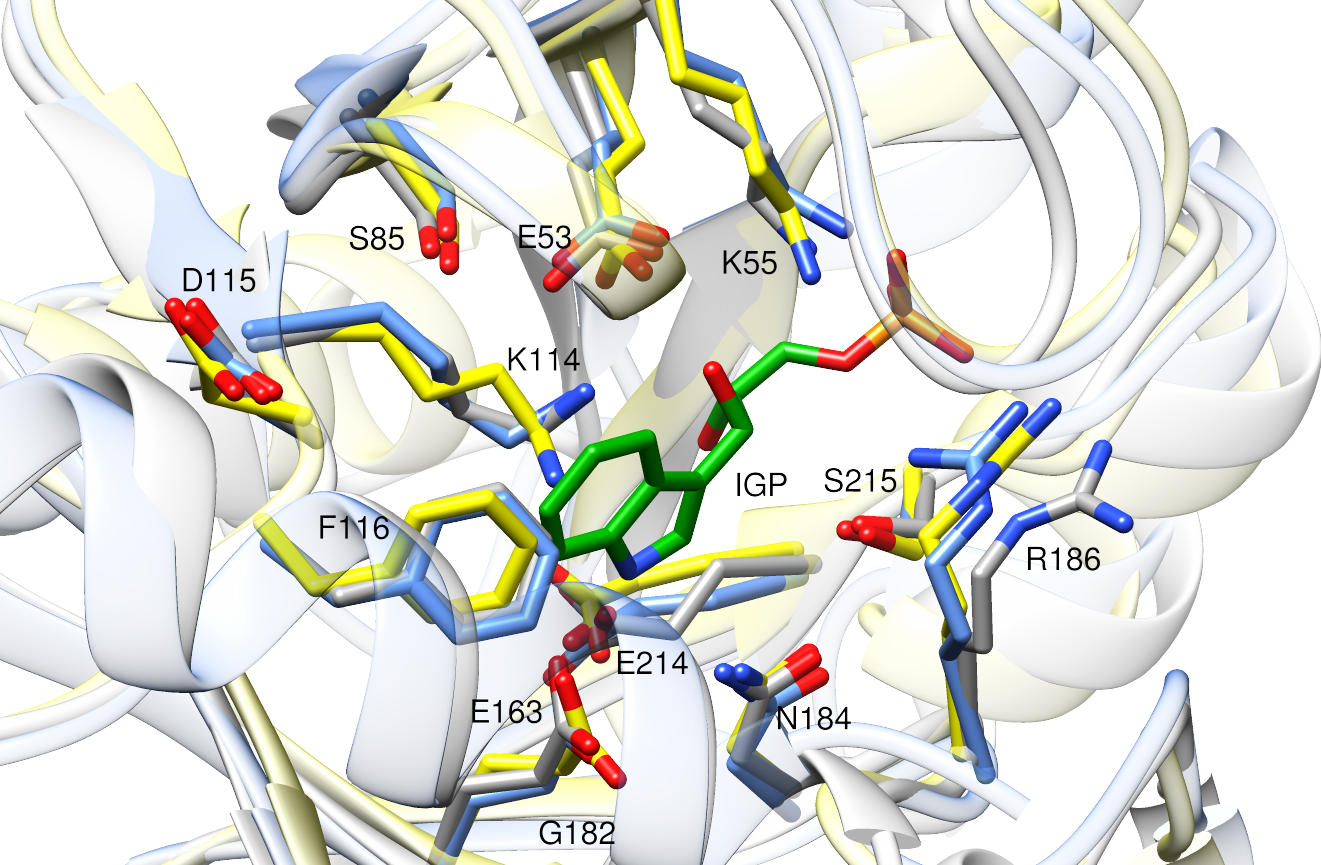


**Fig S2. Aligned Chemical Signature residues for KGPDC with bound d-ribulose 5-phosphate (5RP) in green.** Crystal structures from *E. coli* (PDB 1xbv) in blue and *S. mutans* (PDB 3exr) in yellow. Sequence numbers correspond to *E. coli* structure. The Mg^2+^ ion is rendered as a green sphere. Image rendered in UCSF Chimera [37].


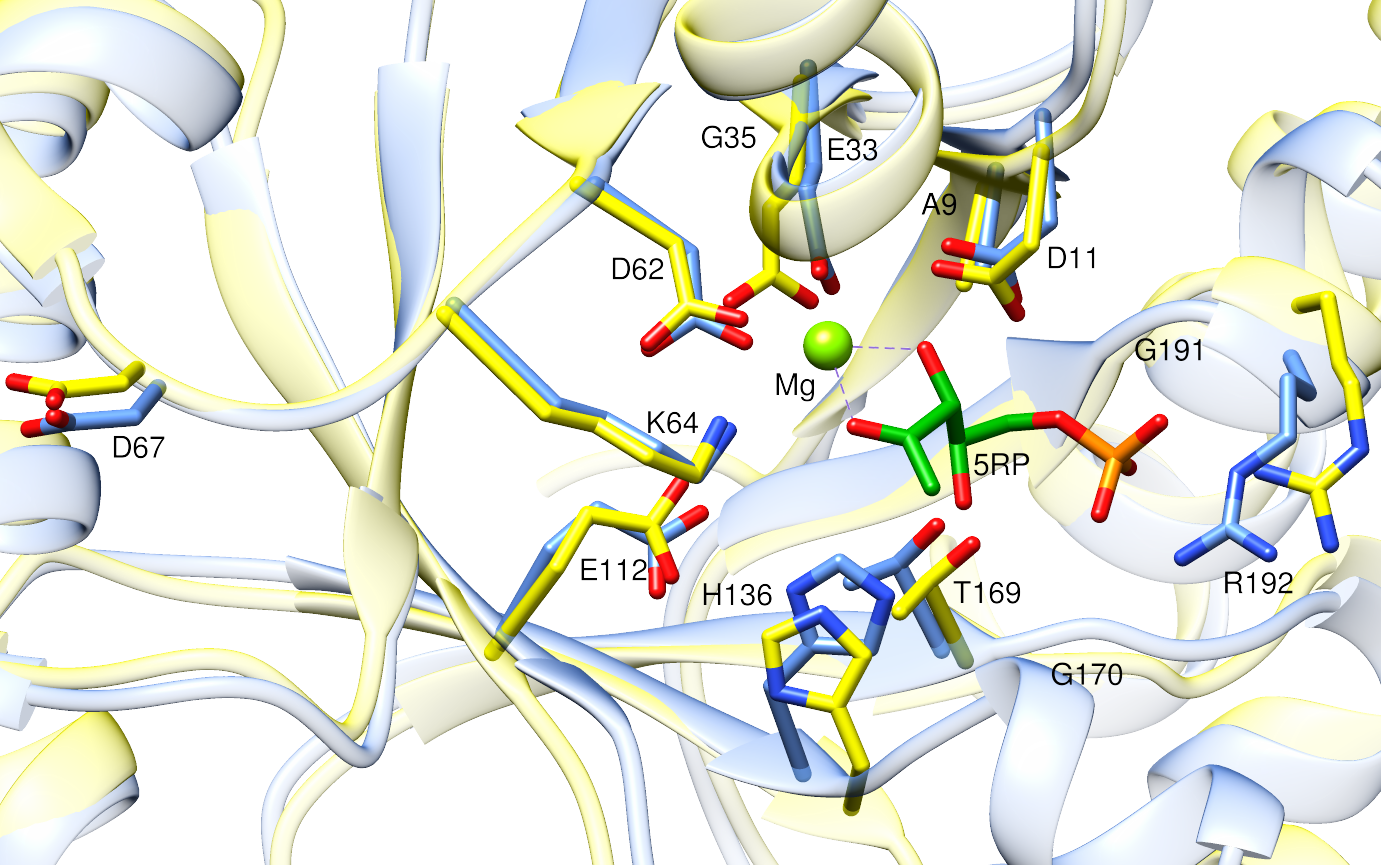


**Fig S3. GRASP-Func clustering of RPBB proteins of known function.** Proteins of known function are represented as nodes. The thickness of each edge shows the degree of similarity between the two connected proteins. PDB IDs: 1pii:N, 1i4n, 2c3z (1a-c, respectively); 1geq, 1qop, 1xc4, 1rd5 (2a-d); 1pii:C, 1lbm (3a-b); 1qo2, 1vzw, 2y85 (4a-c); 1thf, 1h5y, 1ox6 (5a-c); 1rpx, 2fli, 1h1y, 1tqj, 3ovp (6a-e); 1dbt, 1dv7, 1dqw, 1l2u, 2za1, 3qw3, 3l0k (7a-g); 1xbv, 3exr (8a-b); 3ajx, HPS1 (9a-b).

**
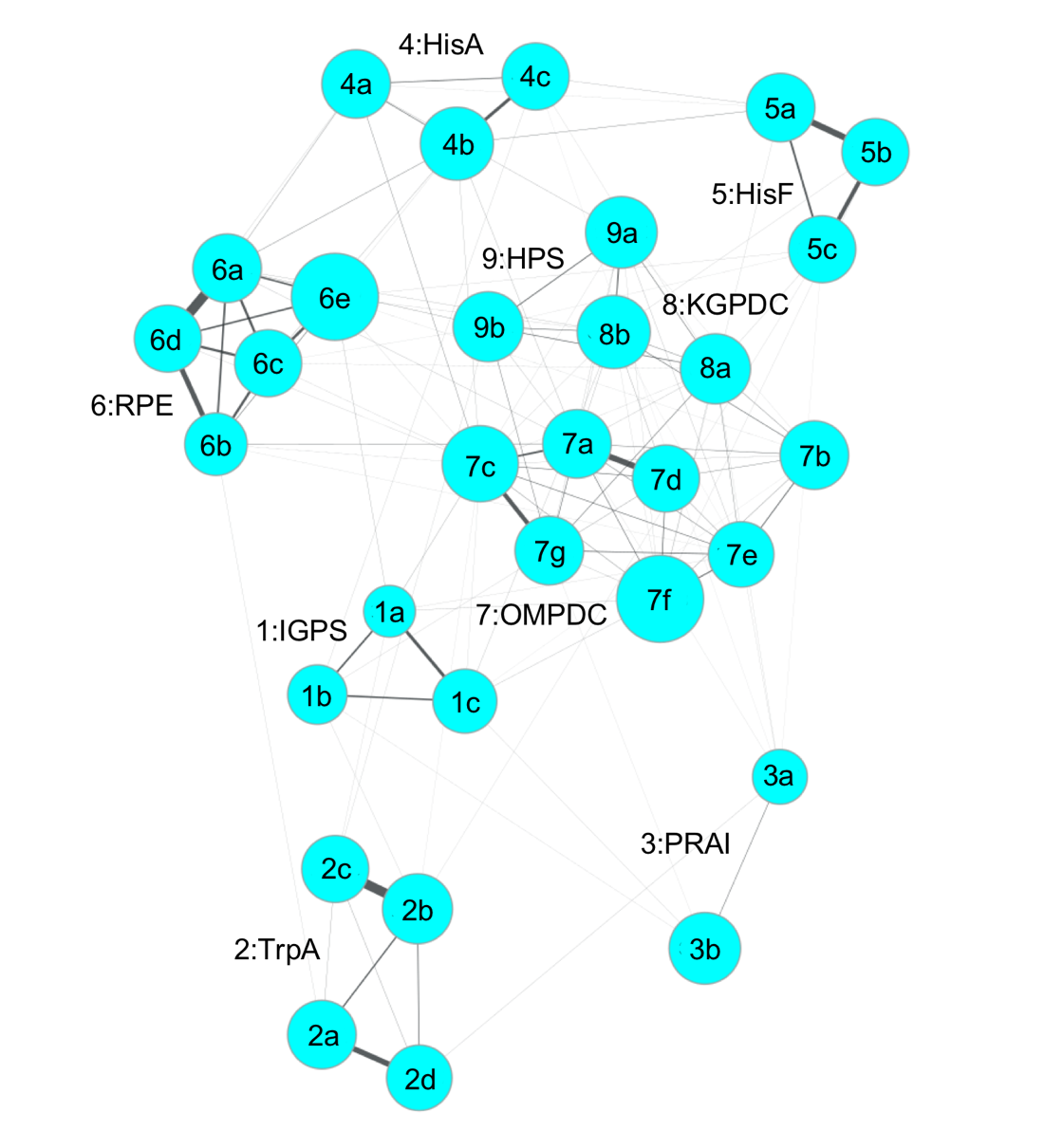
**

**Fig. S4.** **GRASP-Func clustering of 6-HG proteins of known function.** PDB IDs for proteins of known function are represented as nodes, where numbers preceding the PDB ID correspond to functional family designations. The thickness of each edge shows the degree of similarity between the two connected proteins. PDB IDs: 1gai, 1ayx, 1lf9, 1ug9 (1a-d); 3qt9, 3qsp (2a-b); 1cem, 1wu4, 1v5c, 1h12 (3a-d); 1clc, 1kfg, 1ksc, 1ia6 (4a-d); 2d5j, 2zzr (5a-b); 2okx, 3w5m, ALR1 (6a-c); 4ufc, 2eac, ALF1, ALF2 (7a-d); 2jf4, TRE1 (8a-b); 2d8l (9); 3ren (10); 1v7x, 2cqs, CDP1 (11a-c); 1h54, NGP1 (12a-b); 1fp3, 2gz6 (13a-b).

**
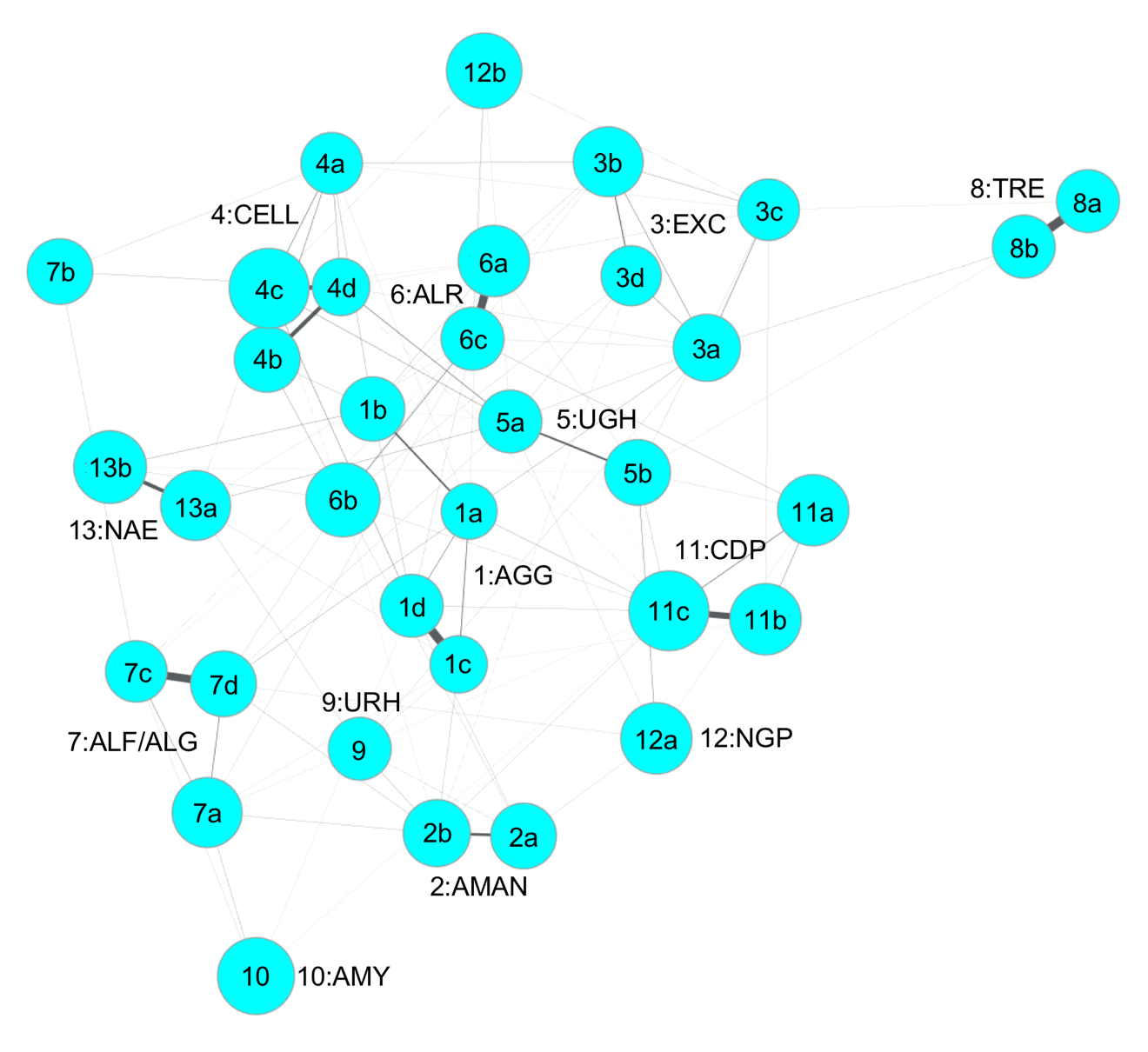
**

**Fig. S5. GRASP-Func clustering of CAL/G proteins of known function.** The PDB IDs for proteins of known function are represented as nodes, where numbers preceding the PDB ID correspond to functional family designations. The thickness of each edge shows the degree of similarity between the two connected proteins. PDB IDs: 1m4w, 1h4g, 1bcx (1a-c); 1uu4, 1h8v, 2nlr (2a-c); 1z3t, 1dy4, 2rfw (3a-c); 2ayh, 1dyp, 3ilf, 2vy0, 1mve (4a-e); 1uai, 1j1t, 1vav (5a-c); 2fir, 1y43 (6a-b).

**
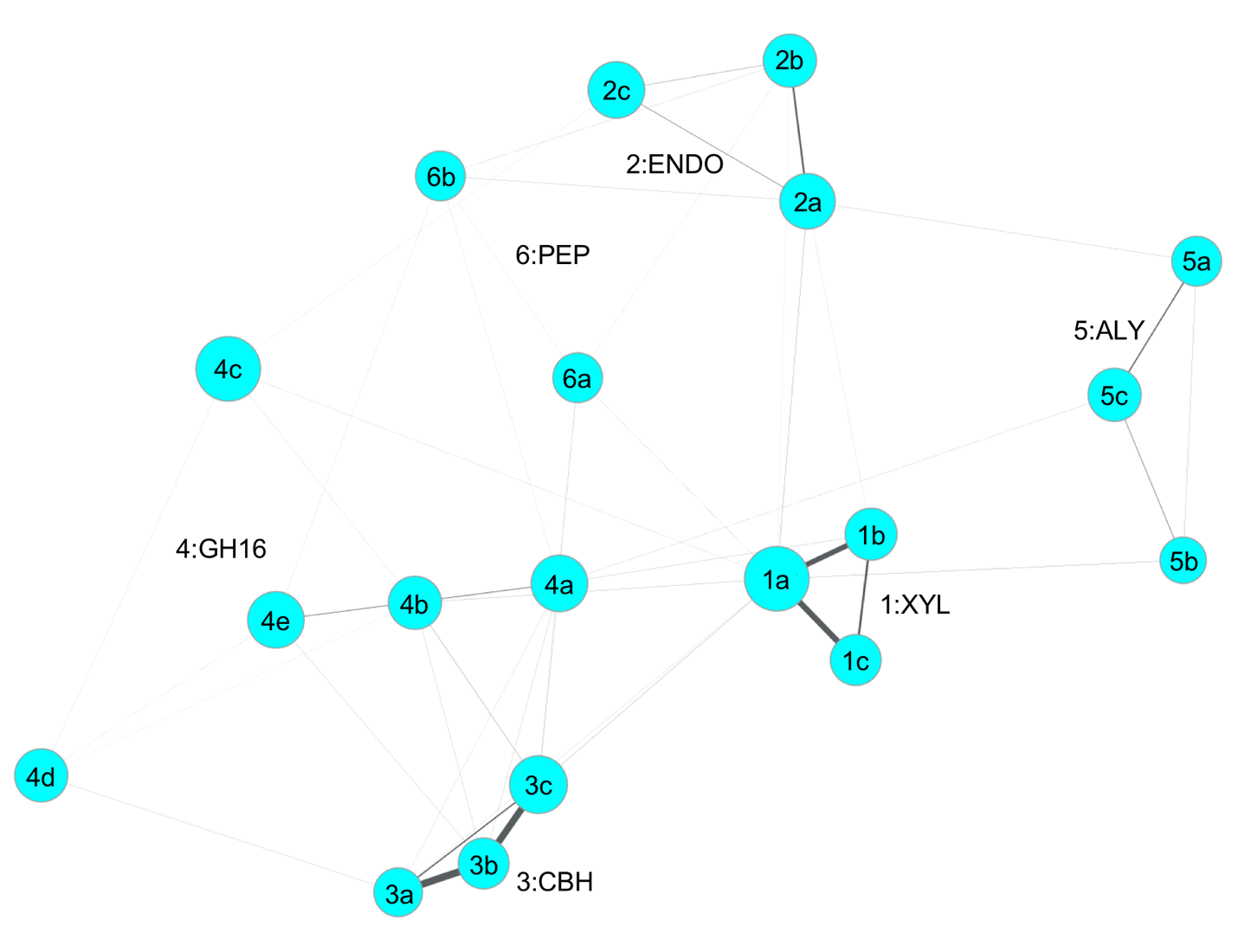
**

**References**

1. Yasueda H, Kawahara Y, and Sugimoto S (1999) *Bacillus subtilis* *yckG* and *yckF* encode two key enzymes of the ribulose monophosphate pathway used by methylotrophs, and *yckH* is required for their expression. J Bacteriol 181(23):7154-7160.

2. Birgisson H, Fridjonsson O, Bahrani-Mougeot FK, Hreggvidsson GO, Kristjansson JK, and Mattiasson B (2004) A new thermostable alpha-L-arabinofuranosidase from a novel thermophilic bacterium. Biotechnol Lett 26(17):1347-1351.

3. Leonard R, Pabst M, Bondili JS, Chambat G, Veit C, Strasser R, and Altmann F (2008) Identification of an Arabidopsis gene encoding a GH95 alpha1,2-fucosidase active on xyloglucan oligo- and polysaccharides. Phytochemistry 69(10):1983-1988.

4. Ishimizu T, Hashimoto C, Takeda R, Fujii K, and Hase S (2007) A Novel alpha1,2-L-fucosidase acting on xyloglucan oligosaccharides is associated with endo-beta-mannosidase. J Biochem 142(6):721-729.

5. Lee JH, Tsuji M, Nakamura M, Nishimoto M, Okuyama M, Mori H, Kimura A, Matsui H, and Chiba S (2001) Purification and identification of the essential ionizable groups of honeybee, *Apis mellifera* L., trehalase. Biosci Biotechnol Biochem 65(12):2657-2665.

6. Lee JH, Saito S, Mori H, Nishimoto M, Okuyama M, Kim D, Wongchawalit J, Kimura A, and Chiba S (2007) Molecular cloning of cDNA for trehalase from the European honeybee, *Apis mellifera* L., and its heterologous expression in *Pichia pastoris*. Biosci Biotechnol Biochem 71(9):2256-2265.

7. Reichenbecher M, Lottspeich F, and Bronnenmeier K (1997) Purification and properties of a cellobiose phosphorylase (CepA) and a cellodextrin phosphorylase (CepB) from the cellulolytic thermophile Clostridium stercorarium. Eur J Biochem 247(1):262-267.

8. Nihira T, Nakai H, Chiku K, and Kitaoka M (2012) Discovery of nigerose phosphorylase from Clostridium phytofermentans. Appl Microbiol Biotechnol 93(4):1513-1522.

9. Wilmanns M, Priestle JP, Niermann T, and Jansonius JN (1992) Three-dimensional structure of the bifunctional enzyme phosphoribosylanthranilate isomerase: indoleglycerolphosphate synthase from *Escherichia coli* refined at 2.0 A resolution. J Mol Biol 223(2):477-507.

10. Knochel T, Pappenberger A, Jansonius JN, and Kirschner K (2002) The crystal structure of indoleglycerol-phosphate synthase from *Thermotoga maritima*. Kinetic stabilization by salt bridges. J Biol Chem 277(10):8626-8634.

11. Schneider B, Knochel T, Darimont B, Hennig M, Dietrich S, Babinger K, Kirschner K, and Sterner R (2005) Role of the N-terminal extension of the (βα)8-barrel enzyme indole-3-glycerol phosphate synthase for its fold, stability, and catalytic activity. Biochemistry 44(50):16405-16412.

12. Yamagata Y, Ogasahara K, Hioki Y, Lee SJ, Nakagawa A, Nakamura H, Ishida M, Kuramitsu S, and Yutani K (2001) Entropic stabilization of the tryptophan synthase alpha-subunit from a hyperthermophile, *Pyrococcus furiosus*. X-ray analysis and calorimetry. J Biol Chem 276(14):11062-11071.

13. Weyand M and Schlichting I (1999) Crystal structure of wild-type tryptophan synthase complexed with the natural substrate indole-3-glycerol phosphate. Biochemistry 38(50):16469-16480.

14. Jeong MS, Jeong JK, Lim WK, and Jang SB (2004) Structures of wild-type and P28L/Y173F tryptophan synthase alpha-subunits from *Escherichia coli*. Biochem Biophys Res Commun 323(4):1257-1264.

15. Kulik V, Hartmann E, Weyand M, Frey M, Gierl A, Niks D, Dunn MF, and Schlichting I (2005) On the structural basis of the catalytic mechanism and the regulation of the alpha subunit of tryptophan synthase from *Salmonella typhimurium* and BX1 from maize, two evolutionarily related enzymes. J Mol Biol 352(3):608-620.

16. Henn-Sax M, Thoma R, Schmidt S, Hennig M, Kirschner K, and Sterner R (2002) Two (βα)(8)-barrel enzymes of histidine and tryptophan biosynthesis have similar reaction mechanisms and common strategies for protecting their labile substrates. Biochemistry 41(40):12032-12042.

17. Lang D, Thoma R, Henn-Sax M, Sterner R, and Wilmanns M (2000) Structural evidence for evolution of the β/α barrel scaffold by gene duplication and fusion. Science 289(5484):1546-1550.

18. Kuper J, Doenges C, and Wilmanns M (2005) Two-fold repeated (βα)4 half-barrels may provide a molecular tool for dual substrate specificity. EMBO Rep 6(2):134-139.

19. Due AV, Kuper J, Geerlof A, von Kries JP, and Wilmanns M (2011) Bisubstrate specificity in histidine/tryptophan biosynthesis isomerase from *Mycobacterium tuberculosis* by active site metamorphosis. Proc Natl Acad Sci U S A 108(9):3554-3559.

20. Banfield MJ, Lott JS, Arcus VL, McCarthy AA, and Baker EN (2001) Structure of HisF, a histidine biosynthetic protein from *Pyrobaculum aerophilum*. Acta Crystallogr D Biol Crystallogr 57(Pt 11):1518-1525.

21. Chaudhuri BN, Lange SC, Myers RS, Davisson VJ, and Smith JL (2003) Toward understanding the mechanism of the complex cyclization reaction catalyzed by imidazole glycerolphosphate synthase: crystal structures of a ternary complex and the free enzyme. Biochemistry 42(23):7003-7012.

22. Kopp J, Kopriva S, Suss KH, and Schulz GE (1999) Structure and mechanism of the amphibolic enzyme D-ribulose-5-phosphate 3-epimerase from potato chloroplasts. J Mol Biol 287(4):761-771.

23. Akana J, Fedorov AA, Fedorov E, Novak WRP, Babbitt PC, Almo SC, and Gerlt JA (2006) d-Ribulose 5-Phosphate 3-Epimerase:  Functional and Structural Relationships to Members of the Ribulose-Phosphate Binding (β/α)8-Barrel Superfamily. Biochemistry 45(8):2493-2503.

24. Jelakovic S, Kopriva S, Suss KH, and Schulz GE (2003) Structure and catalytic mechanism of the cytosolic D-ribulose-5-phosphate 3-epimerase from rice. J Mol Biol 326(1):127-135.

25. Wise EL, Akana J, Gerlt JA, and Rayment I (2004) Structure of D-ribulose 5-phosphate 3-epimerase from *Synechocystis* to 1.6 A resolution. Acta Crystallogr D Biol Crystallogr 60(Pt 9):1687-1690.

26. Liang W, Ouyang S, Shaw N, Joachimiak A, Zhang R, and Liu ZJ (2011) Conversion of D-ribulose 5-phosphate to D-xylulose 5-phosphate: new insights from structural and biochemical studies on human RPE. FASEB J 25(2):497-504.

27. Appleby TC, Kinsland C, Begley TP, and Ealick SE (2000) The crystal structure and mechanism of orotidine 5'-monophosphate decarboxylase. Proc Natl Acad Sci U S A. 97(5):2005-2010.

28. Wu N, Mo Y, Gao J, and Pai E (2000) Electrostatic stress in catalysis: structure and mechanism of the enzyme orotidine monophosphate decarboxylase. Proc Natl Acad Sci U S A 97(5):2017-2022.

29. Miller BG, Hassell AM, Wolfenden R, Milburn MV, and Short SA (2000) Anatomy of a proficient enzyme: the structure of orotidine 5'-monophosphate decarboxylase in the presence and absence of a potential transition state analog. Proc Natl Acad Sci U S A 97(5):2011-2016.

30. Harris P, Poulsen JN, Jensen K, and Larsen S (2002) Substrate binding induces domain movements in orotidine 5'-monophosphate decarboxylase. J Mol Biol 318(4):1019-1029.

31. Tokuoka K, Kusakari Y, Krungkrai SR, Matsumura H, Kai Y, Krungkrai J, Horii T, and Inoue T (2008) Structural basis for the decarboxylation of orotidine 5'-monophosphate (OMP) by *Plasmodium falciparum* OMP decarboxylase. J Biochem 143(1):69-78.

32. French JB, Yates PA, Soysa DR, Boitz JM, Carter NS, Chang B, Ullman B, and Ealick SE (2011) The *Leishmania donovani* UMP synthase is essential for promastigote viability and has an unusual tetrameric structure that exhibits substrate-controlled oligomerization. J Biol Chem 286(23):20930-20941.

33. Heinrich D, Diederichsen U, and Rudolph MG (2009) Lys314 is a nucleophile in non-classical reactions of orotidine-5'-monophosphate decarboxylase. Chemistry 15(27):6619-6625.

34. Wise EL, Yew WS, Akana J, Gerlt JA, and Rayment I (2005) Evolution of enzymatic activities in the orotidine 5'-monophosphate decarboxylase suprafamily: structural basis for catalytic promiscuity in wild-type and designed mutants of 3-keto-L-gulonate 6-phosphate decarboxylase. Biochemistry 44(6):1816-1823.

35. Li GL, Liu X, Nan J, Brostromer E, Li LF, and Su XD (2009) Open-closed conformational change revealed by the crystal structures of 3-keto-L-gulonate 6-phosphate decarboxylase from *Streptococcus mutans*. Biochem Biophys Res Commun 381(3):429-433.

36. Orita I, Kita A, Yurimoto H, Kato N, Sakai Y, and Miki K (2010) Crystal structure of 3-hexulose-6-phosphate synthase, a member of the orotidine 5'-monophosphate decarboxylase suprafamily. Proteins 78(16):3488-3492.

37. Pettersen EF, Goddard TD, Huang CC, Couch GS, Greenblatt DM, Meng EC, and Ferrin TE (2004) UCSF Chimera--a visualization system for exploratory research and analysis. J Comput Chem 25(13):1605-1612.
